# Acknowledgement to Reviewers of *Molecules* in 2016

**DOI:** 10.3390/molecules22010113

**Published:** 2017-01-11

**Authors:** Molecules Editorial Office

**Affiliations:** MDPI AG, St. Alban-Anlage 66, 4052 Basel, Switzerland; molecules@mdpi.com

The editors of *Molecules* would like to express their sincere gratitude to the following reviewers for assessing manuscripts in 2016.

We greatly appreciate the contribution of expert reviewers, which is crucial to the journal’s editorial process. We aim to recognize reviewer contributions through several mechanisms, of which the annual publication of reviewer names is one. Reviewers receive a voucher entitling them to a discount on their next MDPI publication and can download a certificate of recognition directly from our submission system. Additionally, reviewers can sign up to the service Publons (https://publons.com) to receive recognition. Of course, in these initiatives we are careful not to compromise reviewer confidentiality. Many reviewers see their work as a voluntary and often unseen part of their role as researchers. We are grateful to the time reviewers donate to our journals and the contribution they make.

If you are interested in becoming a reviewer for *Molecules*, see the link at the bottom of the webpage http://www.mdpi.com/reviewers.

The following reviewed for *Molecules* in 2016:
Abarbri, MohamedHu, GuochangPereira, VandaAbdi, SalahadinHu, JiangPerera, Omaththage P.Abe, SumikoHu, MingKuanPeressini, DonatellaAbenavoli, LudovicoHu, PingPeretz, AviAbitbol, TiffanyHu, Teh-minPerez, JoelmaAbourashed, EhabHu, XinzhongPerez, LarkAbraham, E. M.Hu, YinlingPérez, Andy J.Abriata, Luciano A.Huang, A-meiPerez-Jimenez, JaraAbuelo, ÁngelHuang, Chi-ChangPérez-Lamela, ConcepciónAbu-Reidah, Ibrahim M.Huang, Chih-ChingPerez-Stable, CarlosAcero, N.Huang, Chun-YungPerić, MihaelaAckerman, H. C.Huang, FeihePericàs, Miquel A.Acosta, EdgarHuang, Hsi-YaPerrone, GiancarloAdemiluyi, Adedayo OluwaseunHuang, Hsu-ShanPerrotti, NicolaAfaq, FarrukhHuang, Hui-ChiPerrow, KaraAfonso, Clélia N.Huang, Hui-PeiPerticone, FrancescoAgerbirk, NielsHuang, JianguoPeruch, FrédéricAgrawal, PrashansaHuang, Jiann-JyhPescitelli, GennaroAgudelo-Romero, PatriciaHuang, Liang-FengPéter, AntalAguilar, AlfredoHuang, Lin-FangPeterson, JuliaAhmad, NihalHuang, LingPeterson, LarrynAhmed, NisarHuang, RenhuaPetitjean, AnneAhn, Mok-RyeonHuang, Ren-YeongPetretto, Giacomo L.Ahn, SangdooHuang, Sheng-YouPetriccione, MilenaAiello, FrancescaHuang, Tzou-ChiPetroianu, Georg A.Aires, AlfredoHuang, WeiPetruzzi, LeonardoAkhavan, OmidHuang, Wei-JanPetzer, Jacobus P.Akhmedov, Novruz G.Huang, Wen-ChungPeyron, Jean-FrançoisAkitsu, TakashiroHuang, Wen-HsinPezzani, RaffaeleAlabugin, I.Huang, WenlinPfrengle, FabianAlauddin, Mian MHuang, Ya-lingPhilipp, BodoAlbini, AngeloHuang, YoujuPhilips, NeenaAl-Dujaili, EmadHuang, ZhiPhillips, DavidAlexandra M. M., AntunesHubicka, UrszulaPhillips, Robert S.Alexandre-Gouabau, Marie-cécile F.Hübner, FlorianPi, RongbiaoAlexiou, ChristophHuc, IvanPicariello, GianlucaAl-Horani, RamiHuczyński, AdamPichler, MartinAlikhani, EsmailHuefner, AntjePicone, GianfrancoAllais, FlorentHueso, Jose L.Piersanti, GiovanniAllen, JeremyHuh, Yong-MinPieters, LucAller, PatricioHui, Kam M.Pietrucci, FabioAllwood, Daniel M.Hullar, MeredithPigge, F. ChristopherAlmajano, María PilarHulme, AlisonPignitter, MarcAlmansa, CarmenHulme, ChristopherPillay, VinessAlmerico, Anna MariaHung, Wei HsuanPina, Cristina DellaAltomare, CosimoHunt, Piper ReidPinal, RodolfoAlvarenga, ElsonHunter, LukePineda, TeresaÁlvarez, MercedesHunter, RogerPiñeiro Gomez, MartaAlvarez Ortí, ManuelHuo, Fang JunPino-Figueroa, AlejandroAlvarez-Lorenzo, CarmenHuq, LailaPiñol, MilagrosAlvarez-Suarez, Josè MiguelHussein, Ahmed A.Pinto, Diana C. G. A.Alves, ElianaHutchens, Michael P.Pinto, Joana Isabel MonteiroAlway, Stephen E.Hutton, CraigPiosik, JacekAmarowicz, RyszardHwang, Bang YeonPires, E.Ambrogi, ValeriaHwang, In KooPires, JoãoAmiche, MohamedHwang, Kyung-RanPiro, BenoitAmorós, AsunciónHwang, Thomas I-ShengPisabarro, Antonio G.Amouri, HaniHwu, Jih-RuPischetsrieder, MonikaAmslinger, SabineHymel, DavidPittelkow, MichaelAn, Hyun JooHyun, Myung HoPitucha, MonikaAn, JiaIacomini, MarcelloPiva, TerrenceAnas, Andrea RoxanneIafisco, MichelePiwowar, AgnieszkaAndersen, Grethe NeumannIannazzo, DanielaPiwowarski, Jakub P.Anderson, Anne J.Iannuccelli, ValentinaPizzeghello, DiegoAnderson, Robin C.Iatrou, HermisPizzimenti, StefaniaAndrade, PaulaIbarra, DavidPlanas, MartaAndreadou, IoannaIbrahem, IsmailPlano, DanielAndreana, PeterIentile, RiccardoPla-Quintana, AnnaAndreoli, MirkoIgarashi, KiharuPlourde, GuyAndreoli, RobertaIglesias, Luis E.Pniewski, TomaszAndrianasolo, EricIglesias, Maria Del RosarioPoddel’sky, Andrey I.Anes, ElsaIkai, TomoyukiPogoń, KrystynaAngelini, PaolaIkegaya, NaokiPohl, PawelAngelino, DonatoIkushiro, Shin-ichiPoirier, Raymond A.Angelone, TommasoIliopoulos, KonstantinosPojman, John A.Angulo, JavierIlisz, IstvánPolavarapu, LakshminarayanaAnna, Vallverdú-QueraltIlton, Eugene S.Poleszak, EwaAnne, LaurençonImperatore, ConcettaPolito, LetiziaAnnese, CosimoInagaki, HidetoshiPollier, JacobAntal, Diana SimonaInami, KeikoPolyak, StevenAntalick, GuillaumeIniesta, JesusPoma, PaolaAntoine, Marie-HélèneInman, SharonPomerantz, William C. K.Anton, SteveInokuchi, TsutomuPons, AntoniAntonchick, AndreyInoue, MasayukiPons, JosefinaAntoniotti, SylvainInoue, ShigeyoshiPontiki, EleniAntunes, LusâniaInserte, JavierPopat, AmiraliAnzai, Jun-ichiIoannou, EfstathiaPopa-Wagner, AurelAojula, Harmesh S.Iohara, DaisukePopiołek, ŁukaszAoki, WataruIorizzi, MariaPopov, AnatoliyAoki, YoshitsuguIrakli, MariaPöppler, Ann-ChristinApone, FabioIsaza Martínez, José HipólitoPoriel, CyrilAppendino, GiovanniIsemura, MamoruPorter, JohnAprea, EugenioIshida, ShintaroPotamitis, ConstantinosAragones, GerardIshikura, MinoruPotapov, AndreiArai, Midori A.Ishola, Mofoluwake M.Potterat, OlivierArai, TakayoshiIslam, Md SorifulPoulsen, Thomas B.Aranda, AgustínIsmaili, LhassanePoupon, ErwanAraniti, FabrizioIsman, MurrayPoussard, LoïcArgyriou, AnagnostisIsman, Murray B.Pozsgai, GaborArgyropoulou, AikateriniIsshiki, YoshiakiPrado, SoizicArias, Renée S.Ita, KevinPranantyo, DickyAriga, KatsuhikoIto, SatoshiPrante, OlafArmanini, DecioIto, ShingoPrasad, SahdeoArmenta, SergioIto, TakeruPrat, MariaArmitage, Ruth AnnIto, TakuyaPratsinis, HarrisArnason, JohnIwaizumi, Masakazu GPreti, DeliaArnhold, JuergenIwamori, MasaoPrice, Joshua L.Arnold, EvaIwaoka, MichioPrieto, MariaArrick, DeniseIwasaka, MasakazuPrieto Garcia, Jose M.Arru, LauraIwasaki, HironoriProestos, CharalamposArterburn, Jeffrey B.Iwasaki, TakashiProf. Dr. Martens, JürgenArulselvan, PalasamyIzawa, HironoriProhens, RafelArvapally, Ravi KumarJaakola, LauraProschak, EwgenijAsai, AkiraJablonski, Dr. Monica M.Pruvost, AlainAsai, DaisukeJablonski, MiroslawPsarra, Anna-maria G.Asai, TeigoJacob, ClausPsomas, GeorgeAshida, HitoshiJacobsen, Elisabeth EgholmPtaszek, AnnaAskalan, RandJaffrezic-Renault, NicolePu, Ying-ChihAsuero, Agustín G.Jäger, MartinPucci, AndreaAta, AtharJagerovic, NadinePuddu, Paolo EmilioAtkins, William M.Jagiello, KarolinaPuglisi, AlessandraAtluri, Venkata S.R.Jain, RachitPulati, NuerxidaAttard, ThomasJaki, BirgitPum, DietmarAtzori, LuigiJakočiūnas, TadasPunchard, Neville AAubert, EmmanuelJames, SametPunta, CarloAvarvari, NarcisJan, Tong RongPütsep, KatrinAvato, PinarosaJandrić, ZoraQian, Bing-JunAverbeck, DietrichJang, Dae SikQian, MingxingAv-Gay, YossefJang, Ik-SoonQin, BolinAwual, Md. RabiulJang, JaehyukQin, WenxinAzémaa, JoëlleJankowska-Anyszka, MarzenaQiu, DeyouAzerad, RobertJansa, JosefQiu, HongdengAznauryan, MikayelJansen, RolfQiu, Ming-huaAzqueta, AmayaJanusz, GrzegorzQuadrelli, PaoloAzuma, KazuoJaque, PabloQuan, GuoxingAzzimonti, BarbaraJara-Palacios, M. JoséQuave, CassandraBabu, DineshJardine, AnwarQueiroz, Maria-João R. P.Bach, HoracioJarstfer, Michael BruceQueiroz Monteiro, RobsonBachawala, PraveenJasinski, MarcinQuesenberry, Peter J.Back, KyoungwhenJasiński, RadomirQuignard, FrançoiseBácskay, IldikóJastrzebska, BeataQuina, FrankBączek, TomaszJászay, ZsuzsaQuintard, Jean-PaulBadu, Shyam R.Javadi, AliQuintavalla, AriannaBaes, MyriamJayant, Rahul DevQuirino, RafaelBaeza, AlejandroJean, LudovicQvit, NirBafor, EnitomeJee, Jun-GooRabanal, FrancescBai, GangJelen, HenrykRace, PaulBai, Shi-QiangJenett-Siems, KristinaRachek, LyudmilaBai, ShuhuaJenssen, HåvardRachwalski, MichałBailey-Kellogg, ChrisJeon, SongheeRadacsi, NorbertBajerska, JoannaJeong, Jin BooRades, ThomasBajpai, RichaJeong, Lak ShinRadić, GordanaBakare, OladapoJeong, Rae-DongRádis-Baptista, GandhiBakker, FT (Freek)Jeong, Seon-YongRadons, JurgenBalawender, RobertJeong, YoonhwaRaes, KatleenBaláž, MatejJeons, SangminRafael Ruiz, Jose´Balemba, Onesmo B.Jerković, IgorRaffa, DemetrioBálint, ErikaJer-Yiing, HoungRaffaele, FrazziBallerini, ClaraJeschke, GunnarRagauskas, Art J.Ballester, Pedro J.Jesionowski, TeofilRagno, RinoBallesteros, AntonioJeszka-Skowron, MagdalenaRahimi, FaridBan, Hyun SeungJetten, AntonRaj, Madhwa H.G.Banach, MarcinJha, MukundRajam, GowrisankarBanc, AmelieJi, BinRajaputra, PallaviBanc, AmélieJi, DebinRakitin, OlegBanerjee, AditiJia, Yi-XiaRakotondraibe, L.H.Banerjee, Hirendra NathJiang, ChangyunRallis, MichailBanfalvi, ZsofiaJiang, ChaoyangRamalho, João P. PratesBankova, VassyaJiang, DeliRamalho, TeodoricoBannister, ThomasJiang, K. E.Ramalingam, LathaBannwarth, Markus B.Jiang, Shu-YeRamidi, PunnamchandarBanskota, Arjun H.Jiang, ZhiyongRamos, SoniaBanti, Christina N.Jiménez Blanco, José L.Ramos-Ortiz, GabrielBao, JieJiménez-Araujo, AnaRamozzi, RomainBao, JinkuJimeno, CirilRamsay, RonaBao, YongpingJin, JiefuRamsden, LawrenceBaran, Timothy M.Jin, TienanRan, ChongzhaoBarba, FranciscoJing, JingRandall, Stephen K.Barbieri, GiuseppeJiráček, JiříRangnekar, AbhijitBarbosa, OveimarJohansen, Steinar D.Rao, ShashaBarbosa Pereira, LetriciaJohansson, Mikael P.Raposo, Maria Filomena JesusBarcelos, GustavoJohler, SophiaRapposelli, SimonaBarclay, ThomasJohn, VargheseRashidinejad, AliBarone, JustinJolkkonen, JukkaRasmuson, ÅkeBarraja, PaolaJones, Jessica L.Rasmussen, HelgeBarrajón-Catalán, EnriqueJones, SimonRateb, Mostafa E.Barraud, NicolasJones, WilliamRatovitski, EdwardBarreira, João C.M.Jonnalagadda, Sreekantha BabuRatz-Łyko, AnnaBarreiro, Sonia LosadaJonnalagadda, Subash C.Rautio, JarkkoBarrio, PabloJordao, Antonio M.Ravelli, DavideBarron, Andrew R.Jørgensen, KåreRawel, HarshadraiBarroso-Bogeat, AdriánJoshi, BharatRay, Dr SudipBartolini, ManuelaJoule, JohnRay, UdayanBartolomé, BegoñaJuan, M. EmíliaRaynal, MatthieuBartolotti, MassimoJuang, Tzong-YuanRebroš, MartinBasco, Leonardo K.Juarranz, ÁngelesReddy, Narsa M.Basiak, EwelinaJugé, SylvainReese, R. NeilBasini, GiuseppinaJullian, CarolinaRega, NadiaBasso, AndreaJung, Byung HwaRehrig, Erin M.Basso, DanielaJung, Ho-SupReiger, MatthiasBattino, MaurizioJung, Sang HunReis, Catarina PintoBaudoux, JérômeJung, SeunhoReiser, OliverBauer, TomaszJung, Young-SukReisman, Scott A.Baum, ChristelJunier, PilarRekka, Eleni A.Bavaresco, LuigiJunior, Walter A. RomanRemião, FernandoBayer, Ilker S.Jurczak, JanuszRemko, MilanBayry, JagadeeshJurikova, TundeRen, DachengBazaka, KaterynaJuszczak, LesławRen, JianguoBazylińska, UrszulaJux, NorbertRen, Jun-LiBeall, Gary W.Jyh-fei, LiaoRenaudineau, YvesBeaudoin, ClaudeKachlicki, PiotrRescifina, AntonioBeaudry, ChrisKaczor, AgnieszkaResende Maldonado, RafaelBeauvais, AnneKadam, ShekharRevilla, EugenioBeberok, ArturKadokawa, Jun-ichiReyes, FernandoBeck, AndreasKador, LotharReynisson, JóhannesBedini, EmilianoKadota, IsaoRhee, Young HoBednarczyk-Cwynar, BarbaraKahlert, StefanRho, Jung-RaeBednarska, KatarzynaKaipa, UshasreeRicelli, AlessandraBednarski, PatrickKakkar, Ashok K.Richardson, AlanBeier, Ross C.Kalinowska-Lis, UrszulaRiela, SerenaBeld, JorisKalinowski, Danuta S.Riemann, MaxBelfield, Kevin D.Kalska-Szostko, B.Riesen, HansBelfrage, Anna KarinKamdem, D. PascalRigano, DanielaBelide, SrinivasKamiloglu, SenemRijo, PatríciaBelitsky, Jason M.Kanai, YoshikatsuRimondini, LiaBell, Thomas W.Kanakkanthara, ArunRinge, JochenBellés, José MaríaKanchiswamy, Chidananda NagamangalaRinner, UweBello, Nicholas T.Kanda, YasuharuRío Segade, SusanaBełtowski, JerzyKang, ChanKyuRioual, S.Benaglia, MaurizioKang, ChulhunRiu-Aumatell, MontserratBencharit, SompopKang, Dong-KuRiva, RenataBenelli, GiovanniKang, Jae SeungRizzi, FedericaBenito, José ManuelKang, Jihee LeeRIzzo, Angela MariaBenito, SantiagoKang, Jong SeongRizzuti, BrunoBenson, HeatherKang, JonghoonRobert, Jean-MichelBenzeroual, Kenza E.Kang, Jun YongRobert, MitchellBeppu, FumiakiKang, SinyoungRoberto, DominiqueBeretta, GiangiacomoKang, SORoberts, Sue A.Bergbreiter, David EKang, Sun ChulRobertson, Mark J.Bergens, StevenKanie, OsamuRobien, WolfgangBerger, Ralf GKankanala, JayakanthRobledo, Sara M.Bergès, ThierryKano, MitsuyoshiRoca-Sanjuán, DanielBerhow, MarkKao, Chai-LinRocco, AnnaBerlicki, ŁukaszKapalavavi, BrahmamRochais, ChristopheBerlin, K. DarrellKappel, ChristianRoche, ManonBernal, ClaudiaKapusta-Duch, JoannaRodionov, RomanBernard, DenzilKarafiloglou, PadeleimonRodrigues, RafaelBernardi, LucaKaralekas, DimitrisRodriguez, JoseBernhardt, Harold S.Karamanis, PanaghiotisRodríguez, JaimeBernhoft, AkselKaramaouna, FilitsaRodríguez, MarioBernini, RobertaKarczmarczyk, AleksandraRodríguez Herrera, RaúlBerrie, CindyKärkönen, AnnaRodriguez-Cabezas, Maria ElenaBerta, GraziellaKarlic, HeidrunRodríguez-Dafonte, PedroBerti, FedericoKarmakar, ParthaRodriguez-Estrada, Maria TeresaBertinaria, MassimoKarnaouri, AnthiRodríguez-Franco, María IsabelBertolini, JosephKaroglan, MarkoRodríguez-López, JuliánBérubé, GervaisKarpoormath, RajshekharRoelants, KimBesada, CristinaKarsili, TolgaRogelj, SneznaBesalú, EmiliKaschula, Catherine H.Rogers, Robin D.Besson, ThierryKashfi, KhosrowRogozhin, E.A.Bethanis, KostasKashiwada, YoshikiRoh, ChanghyunBeuerle, FlorianKashman, YoelRojo, JavierBezdetnaya, LinaKatagiri, HiroshiRolan, PaulBezirtzoglou, EugeniaKataoka, H.Rolfe, Barbara E.Bhagavathy, Ganga ViswanathanKataoka, HiromiRolim, LarrisaBhattacharya, ChandrabaliKataoka, TakaoRollini, ManuelaBhosale, SheshanathKatayama, ShigeruRomalde, JesusBhutoria, SavitaKath-Schorr, StephanieRomero, AlejandroBi, KaishunKathuria, HimanshuRomero, Marta R.Biagini, GiuseppeKatiyar, Santosh K.Romero Álvarez, IrmaBianchi, AntonioKato, AtsushiRosa, AntonellaBianco, Armando DorianoKato, KoichiRose, DevinBiava, MariangelaKato, MasayaRosende, Eugenia GonzálezBiavatti, Maique W.Katoh, TadashiRosenmann, HannaBiddle, AdrianKatsila, TheodoraRosini, MichelaBiedermann, DavidKatsuzaki, HirotakaRoss, SamirBijak, MichałKatuchova, JanaRoss, Samir A.Billard, ThierryKaufman, Teodoro S.Rossi, MiriamBills, GeraldKaufmann, DorotheaRossner, PavelBimbo, Luis M.Kauppinen, AnuRotblat, BarakBirch, JohnKavallieratos, Nickolas G.Rotureau, PatriciaBishayee, AnupamKawabata, KyuichiRoudier, EmilieBishop, Anthony C.Kawahara, TeppeiRousseau, G.Bishop-Hurley, SharonKawai, YoshichikaRoussis, VasileiosBisignano, GiuseppeKawakami, SusumuRoutier, SylvainBisti, SilviaKawakita, HidetakaRoy, ReneBisztray, Gyoergy D.Kawamura, AkiraRoy, SubhadeepBjørndal, BodilKawamura, FumioRóżalska, BarbaraBlair, JessicaKawao, NaoyukiRubino, FedericoBlais, AnneKawashima, HidekiRubiolo, PatriziaBlakely, RandyKawashima, TakayukiRucker, Robert B.Blanco, Rosa M.Kay, Colin D.Rudick, JonathanBłaszczak-Świątkiewicz, KatarzynaKay, GraemeRudkowska, IwonaBłażewska, Katarzyna M.Kayaki, YoshihitoRudyk, OlenaBlazquez, M. AmparoKelesidis, TheodorosRuelland, EricBloem, ElkeKelly, Ryan T.Ruggerone, PaoloBoa, Andrew N.Kerr, William L.Rühl, MartinBobe, GerdKeserű, György M.Ruiz, JoséBoddy, Christopher N.Keski-Rahkonen, PekkaRuiz, T.Boechat, NubiaKeski-Saari, SaritaRuiz-Aceituno, LauraBoege, FritzKessel, DavidRuiz-Gómez, GloriaBoeglin, AlexKessel, LineRulisek, LubomirBoga, CarlaKeul, HelmutRuparel, ShivaniBöhm, VolkerKeum, GyochangRusso, MariaBohn, TorstenKeum, Young SooRusso, MarinaBoitel-Conti, MichèleKevil, Christopher GRuthstein, SharonBojarski, AndrzejKeyzers, RobRutjes, Floris P. J. T.Bolibok-Brągoszewska, HannaKhalid, Sami A.Ruzicka, AlesBolivar, JuanKhan, ImranRyan, James G.Bolm, CarstenKhan, MohsinRyffel, BernhardBolognesi, AndreaKhan, Muhammad IqbalRyu, Jong HoonBolotov, LeonidKhenoussi, NabylRyu, Mi HeonBondon, ArnaudKhulbe, K. C.S. M., Malathy SonyBondonno, CatherineKhun, Nay WinS. Silva, M. LuísaBoni, AdrianoKiba, AkinoriSabatier, Jean-MarcBonifácio, VascoKibayashi, KazuhikoSabila, PaulBonilla, C. E. P.Kiec-Kononowicz, KatarzynaSacks, GavinBonnet, VeroniqueKiefer, ChristianeSackus, AlgirdasBoo, Yong ChoolKietzmann, ThomasSacré, Pierre-YvesBorbas, K. EszterKievit, Forrest M.Saczewski, JarosławBordes, PatriciaKikuchi, HaruhisaSada, KazukiBorges, AnabelaKim, Byung-SooSadiq, Muhammad WaqasBorochov-Neori, HamutalKim, Chul YoungSagebiel, John C.Borondo, FlorentinoKim, Chun HoSah, Hong-KeeBorowska, MartaKim, Chu-YoungSaha, MrinmoyBorsani Cambón, Julio OmarKim, Dong-HyunSai Hanuman Sagar, BodduBoryczka, StanisławKim, DongukSaiano, FilippoBosch, JoaquimKim, Gheun-HoSaid, Ahmed M.Botelho, GoretiKim, Han-doSaisho, YoshifumiBotre, FrancescoKim, HyemeeSaito, ShinichiBoulares, HamidKim, Hyung KwounSakakibara, HiroyukiBouquillon, SandrineKim, Hyung SikSakamoto, ToshioBour, PetrKim, Hyun-JoongSakilam, SatishBousbaa, HassanKim, HyunwooSala, RobertoBouvier, GuillaumeKim, Jae YoungSaladino, RaffaeleBowden, BruceKim, JaheonSalafranca Lázaro, Francisco J.Bowling, LeeKim, Ji YeonSalahub, DennisBowman, MichaelKim, Jin-KyungSalazar-Olivo, LuisBoyd, BenjaminKim, Jong-HoSaleem, MohammadBoyle, Joseph J.Kim, Joong SunSaleh, Mahmoud A.Bracher, FranzKim, JoungmokSalehi-Reyhani, AliBrachet, EtienneKim, Jung-AeSalgo, Professor AndrasBraga, SusanaKim, JunghyunSaliba, Anthony J.Brain, Susan D.Kim, KikangSalim, VonnyBramley, PeterKim, Kil-SooSalvatore, Ralph NicholasBranca, CarmenKim, Kyu-BongSalvio, RiccardoBranca, FerdinandoKim, Kyung-suSamanidou, VictoriaBranchini, AlessioKim, Myeong OkSamavedi, SatyavrataBrand, D. JacoKim, SejoongSampson, ClareBrandão, Maria Das GraçasKim, Seok-JhinSampson, Nicole S.Bravo, LauraKim, Seung-WhanSan Martin, CarmenBravo López, Susana B.Kim, Sung YeonSanches-Silva, AnaBreaker, RonaldKim, Sung-HoonSanchez, ChristelleBrecker, LotharKim, SunjooSanchez, GoarBreinbauer, RolfKim, Tae KonSánchez, GloriaBreitenfeld Granadeiro, LuizaKim, Tae-JeongSánchez Migallón, AnaBremner, John B.Kim, YangSánchez-García, DavidBrennan, MarianKim, Yeon-JuSánchez-Mata, María De CortesBreydo, LeonidKim, Young SangSánchez-Moreno, ManuelBriceño, GabrielaKim, Young-jaeSánchez-Pernaute, OlgaBrindisi, MargheritaKim, Young-KyoonSancho, TeresaBroderick, Tom L.Kim, YoungmeeSandhu, Jagdeep K.Broisat, AlexisKim, Young-WooSandjo, LouisBrooks, Amanda E.Kim, YujinSanejouand, Yves-HenriBrouard, DannyKim, Yun-baeSangaraju, DewakarBrown, DarrenKimura, Ken-ichiSangireddy, Sasikiran ReddyBrown, LindsayKinaciyan, TamarSanjust, EnricoBrück, ThomasKinder, DavidSansone, AnnaBruckner, ChristianKing, MichaelSansone, ClementinaBruin, Bas DeKinoshita, TakayoshiSantagati, AndreaBrullo, ChiaraKinthada, RamakumarSantangelo, CarmelaBrun, RetoKiparissides, CostasSanti, ClaudioBrunetti, CeciliaKirby, Christopher W.Santilio, AngelaBruni, GiovannaKirsch, Professeur Emérite GilbertSantillo, MichaelBruni, RenatoKirton, Stewart B.Santos, A. GilBrunklaus, GuntherKiss, RobertSantos, AbelBruns, NicoKitagawa, WataruSantos, ClementinaBrusotti, GloriaKitagishi, HiroakiSantos, RoseaneBrycki, BogumiłKitamura, MasanoriSantos, ValentínBrzezinski, RyszardKitazawa, TakafumiSantos, Willy GlenBučar, Dejan-KrešimirKiyama, RyoitiSantulli, GaetanoBuchowicz, W.Klajnert, B.Sarabia, FranciscoBuckley, M.Klaus, SusanneSarandeses, Luis A.Buddle, BryceKlausen, R. S.Sarazin, CatherineBudzisz, ElzbietaKlaveness, JoSarkar, BiprajitBuemi, Maria RosaKlein, ChristianSartillo-Piscil, FernandoBugaut, XavierKlempt, MartinSatake, HonooBunce, Richard A.Klettner, AlexaŠatínský, DaliborBunker, AlexKluepfel, DanielSato, NaoyukiBunz, Uwe H. F.Kluger, RonaldSato, SoshiBuonanno, ManuelaKlüß, JeannetteSato, SotaBurgos Hernández, ArmandoKnölker, Hans-JoachimSatora, PawelBurley, Jonathan C.Knoshaug, EricSaturnino, CarmelaBuron, FrédéricKnupp, GerdSatyal, PrabodhBurow, MeikeKo, Kam MingSaulnier, LucBurris, Kellie P.Ko, Shun-YaoSavoia, DianellaBurton-Freeman, BrittKo, Young TagSayas, EstrellaBurton-Freeman, M.Kobayashi, KazuhiroSbardella, GianlucaBusconi, MatteoKobayashi, MakotoScala, AngelaBushman, ShaunKobayashi, NobuyukiScalenghe, RiccardoBusinaro, RitaKobets, TetyanaScarfato, PaolaButini, StefaniaKobuchi, ShinjiScarso, AlessandroBuxton, DenisKochi, TakuyaSchafleitner, RolandBystrowska, BeataKocsis, BelaSchaich, KarenCaballero, JulioKogure, NoriyukiSchaller, HubertCabedo, NuriaKoh, Hwee LingSchepdael, Ann VanCabral, CéliaKohler, BernSchinkovitz, AndreasCabral, HoracioKoivisto, AriSchirhagl, RomanaCabral, JaydeeKojima-Yuasa, AkikoSchmeda-Hirschmann, GuillermoCadwallader, KeithKojo, ShosukeSchmid, J.Caelles Franch, CarmenKok, Victor C.Schmid, MatthiasCaffarelli, CarloKolb, PeterSchmiderer, CorinnaCaffrey, PatrickKolbert, ZsuzsannaSchmidl, DoreenCafiero, MauricioKolhar, PoornimaSchmidt, BerndCai, LishengKollár, LászlóSchmidt, MartinCai, XianghaiKolodkin-Gal, IlanaSchmidt, SusannCalderone, VincenzoKomeiji, YutoSchmidtke, MichaelaCalhelha, Ricardo C.Komori, KikuoSchmitz, John C.Calogeropoulou, TheodoraKonaté, KiessounSchneeklothJr., John S.Calvete, JuanKong, In-sooSchneider, BerndCalvete, MarioKong, Siu-KaiSchneider, RaphaelCamacho, M. EncarnaciónKönig, BurkhardSchnermann, Martin J.Camara, Amadou K.S.Konkolewicz, DominikScholz, MarkusCámara-Martos, FernandoKooyman, P. J.Scholz-Ahrens, KatharinaCamarasa, María-JoséKopel, PavelSchonbrun, EthanCamele, IppolitoKoppel, KadriSchönherr, HolgerCamins, AntoniKortz, UlrichSchörken, UlrichCammas-Marion, SandrineKoschella, AndreasSchrag, MatthewCampana, RaffaellaKosmrlj, JanezSchroeder, GrzegorzCampbell, ChristopherKosova, KlaraSchuck, AlyssaCampbell, LeeKostakis, IoannisSchulz, Benjamin L.Campos Rosa, JoaquínKostomitsopoulos, Nikolaos G.Schulz, FrankCanas, SaraKotloski, RobertSchulzová, VěraCandiani, GabrieleKotsikorou, EvangeliaSchurig, VolkerCanela, Dr. RamonKoufaki, MariaSchuurhuis, Gerrit J.Canini, AntonellaKoul, OpenderSchwartz, Stanley A.Cannazza, GiuseppeKoulman, AlbertSchwendicke, FalkCao, BinKoundouras, StefanosScognamiglio, MonicaCao, BoKounis, Nicholas G.Scorziello, AntonellaCao, WeiKourkoutas, YiannisScotti, MarcusCao, ZexingKoutentis, PanayiotisSeabra, Amedea B.Capasso, RaffaeleKouvelis, Vassili N.Seca, AnaCapece, AngelaKovacs, LajosSeca, Ana M. L.Capriati, VitoKovács, Ákos T.Seebacher, WernerCarabineiro, Sónia A. C.Kowalewski, MarkusSeebeck, Florian PCaramori, StefanoKowalski, KonradSegarra-Marti, JavierCaramori, Giovanni F.Kowalski, RadosławSeger, ChristophCarbone, AnnaKoziel, JacekSegi, MasahitoCardona, FrancescaKozliak, Evguenii I.Ségui, BrunoCardoso, Susana MKozłowski, HenrykSeifert, KarlheinzCardoso, Susana M.Kozono, DavidSeijas Vázquez, JulioCaricato, MarcoKraaijeveld, KenSekine, KenTaroCarlsson, JensKrajewska, BarbaraSeley Radtke, KathieCarmine, GuarinoKrajka-Kuźniak, ViolettaSelli, ElenaCarotti, AngeloKrálovec, KarelSelma, María V.Carpéné, ChristianKrämer, StefanieSémeril, DavidCarradori, SimoneKraus, BirgitSen, ArundhutiCarraher Jr., Charles E.Krchňák, ViktorSen, SanghamitraCarrasco, HéctorKregel, StevenSendker, JandirkCarraz, MaelleKreis, WolfgangSeo, Jae HongCarregal-Romero, SusanaKren, VladimirSeo, Seung-YongCarreira, MiguelKresh, J. YashaSeo, Woo DuckCarrion, M. DoraKriegsheim, Alex VonSeo, YongbeomCarrubba, AlessandraKries, HajoSeo, Young-SooCarta, FabrizioKuan, Yu-HsiangSeol, Geun HeeCarter, Wayne G.Kubicek, Christian P.Séraphin, DenisCaruso, CalogeroKubíček, VojtěchSergi, ConsolatoCarvajal, MicaelaKubicova, LenkaSerio, AnnalisaCasassa, FedericoKubina, RobertSerpone, NickCascioferro, StellaKubota, NaotoSerra, AngelsCaselli, AlessandroKuca, KamilSerra, ImmacolataCaspi, YaronKucerik, JiriSerra, RaffaeleCasteel, Darren E.Kucuk, IsrafilSerra, StefanoCastilho, Paula C.Kujawski, WojciechSerralheiro, Maria Luísa M.Castñeiras, AlfonsoKumar, AshutoshSerrano, Jose C. E.Castro, Antonio JesusKumar, GauravSerrano, MariaCastro, MariaKumar, NareshServin, Alain L.Casula, GiuliaKumar, UjendraSestili, PieroCatto, MarcoKuno, ToshiyaSeth, DevanshiCavaliere, ChiaraKunz, WolframSethi, GautamCavalli, RobertaKunze, GotthardSetzer, WilliamCeccherini, MariaKuo, Ping-ChungSgarbossa, PaoloCelia García-Alvarez-Coque, MaríaKurakevych, Oleksandr O.Sgarra, RiccardoCefalas, A. C.Kurdowska, Anna K.Sgorbini, BarbaraCerchia, LauraKurokawa, IchiroShah, Dhaval K.Cerdá Martínez Pujalte, BegoñaKus, Piotr MarekShah, DilipCerrada, Maria LuisaKuwahara, MasayasuShahar Yar, MohammadChabert, PhilippeKuzma, LukaszShahbazi, Mohammad AliChae, HeeyoungKuz’min, VictorShaio, Young-jiChai, YifengKvasnica, MiroslavShalin, SaraChaimbault, PatrickKwak, Jong-YoungShangguang, DihuaChalker, Justin M.Kwan, Hiu-yeeShankar, EswarChallinor, Victoria L.Kwatra, DeepShapiro, Bruce A.Chambers, KarenKwon, Il-KeunSharma, AnjaliChamulitrat, WaleeKwon, OranSharma, Arun KChan, Ben C. L.Kwon, Sung WonSharma, BheshChan, Tak HangKwon, Young-WanSharma, ShervenChan, ThomasKwong, Fuk YeeShaw, AnthonyChan, VincentKyasa, Shiva KumarShaw, Pang-ChuiChand, SubhashKyung, Kyu HangShaw, SubrataChang, Chi K.Kyushin, SoichiroShehu, AmardaChang, Chih-JuiLa Motta, ConcettinaSheikh, FarahChang, Chih-WeiLa Regina, GiuseppeSheldon, JulieChang, Chih-YuLaali, KennethShen, JunChang, Chiou LingLaatsch, HartmutShen, QiangChang, Fang-RongLacaille-Dubois, Marie-AlethShen, Tang-LongChang, Hsueh-WeiLaczka, CsillaShen, Xu ShenChang, Hyeun WookLadero, MiguelShen, Ya-ChingChang, Li-ChingLadero Galán, MiguelShen, Yue-maoChang, Long-SenLaemmerhofer, MichaelSheng, RongChang, Sue-joanLago, N. FaginasSherwood, JamesChang, Yao-FengLahav, OriSheu, Joen-RongChang, Yuan-ShiunLahooti, HooshangShi, DaaingChao, Louis KuopingLaJeunesse, Dennis R.Shi, DayongChao, Ming-WeiLam, YulinShi, HuaChao, Pi-YuLamprou, DimitriosShi, Jie-huaChao, YanjieLamuela Raventos, RosaShi, QiangChapoval, Svetlana P.Lan, Chung-YuShiau, Chung-WaiChappell, JosephLan, TianShieh, Chwen-JenCharles, Marie ThérèseLandete, José MariaShih, Wen-LingChatel, GrégoryLane, MajellaShiina, IsamuChatterjee, JhinukLang, Jian-PingShima, SeigoChaturvedi, PankajLange, HeikoShimada, ShigeruChaves-Lopez, ClemenciaLangeveld, HansShimizu, YoheiChavez, FermanLaport, MarinellaShimoda, HiroshiChazot, Paul L.Laquintana, ValentinoShimodaira, ShigetakaChe, Fang-SikLarraneta Landa, EnekoShimoi, NorihiroCheca, AntonioLatortue, Marie C.Shimomura, NorihiroChefetz, BennyLatosińska, Jolanta NataliaShin, Beom SooChemat, FaridLattuada, MarcoShin, Chae-HoChen, Cheng-LungLau, Christopher S.Shin, Dong-SooChen, Chung-YiLau-Cam, Cesar A.Shin, InjaeChen, Chun-JungLaurent, FERRIÉShin, Jae SupChen, Chun-LongLauro, M. R.Shinada, TetsuroChen, DaiqinLavandera, IvanShinn, SaraChen, DajunLavarda, Francisco CarlosShinomiya, KazufusaChen, Fen-ErLavrentovich, Oleg D.Shipley, PaulChen, GaoLaw, Betty Yuen-KwanShiragami, TsutomuChen, GuibingLawen, AlfonsShirai, KeikoChen, Gwo-ShingLazzarato, LorettaShirakami, YoheiChen, HeruLazzaroni, RobertoShirakawa, HitoshiChen, Hsin-ChunLe Grognec, ErwanShirasawa, KentaChen, HuaiqiongLe Hégarat, LudovicShiro, TomoyaChen, HuimeiLe Pape, PatriceShirsath, SagarChen, Hui-yinLe Pogam, PierreShityakov, SergeyChen, Jian-ZhongLeach, KatieShrestha, GajendraChen, Ji-huaLeadbeater, NicholasShukla, Shivendra D.Chen, JinhuiLechtenberg, MatthiasSiggelkow, HeideChen, JinquanLeclercq, GuySikorska, HannaChen, LiLeclère, PhilippeSiligardi, GiulianoChen, MenglinLedoigt, GérardSilipo, AlbaChen, Miaw-LingLee, AdamSilks, Louis A.Chen, Min-HueyLee, Bun YeoulSilva, Ana CChen, MohanLee, Chang HoonSilva, Ana M.g.Chen, Po-YuanLee, Chang-HunSilva, AnjanaChen, QiLee, Che-HsinSilva, Branca M.Chen, Ruei-mingLee, Chin-FaSilva, Daniel VaronChen, ShilinLee, Ching-KuoSilva, FilomenaChen, Shiu-NanLee, Chong-KilSilva, Jorge CarvalhoChen, ShuaiLee, Choon YoungSilva, LuísChen, TianbaoLee, Choong HwanSilva, Luís R.Chen, TongLee, Dong GunSilva, Maria FátimaChen, XiaolanLee, Dong-ChanSilva, Maria FernandaChen, XinLee, DongwonSilva, SóniaChen, YingchunLee, Eun-HeeSilvar, CristinaChen, Yu-KuoLee, Guem-SanSilvério, Sara C.Chen, Yun-WenLee, Hee SoonSilverman, Richard B.Chen, Zheng (Jake)Lee, Hon ManSilvestri, ElenaCheng, Heung-ChinLee, Huei-JaneSilvestri, RomanoCheng, Jing-JyLee, Hwa JinSimanek, EricCheng, JizhongLee, Hye SukSimes, DinaCheng, Juei-TangLee, Jae WookSimirgiotis, Mario J.Cheng, Qing-QingLee, Jae YeolSimmons, KatieCheng, S. S.Lee, JaehwiSimmons, Peter A.Cheng, Shi-yieLee, JeongmiSimonsen, Henrik ToftCheng, SunLee, Jin-KooSimpson, Bradley S.Cheng, XingguoLee, Jiun-hawSimpson, Garth J.Cheng, Yi-XiangLee, JiyounSims, Ian M.Cheng, YongLee, JonghwiSinger, Robert J.Cheng, Yu-WenLee, JongkookSingh, Ashok K.Cheng, ZhiqiangLee, Jong-SeokSingh, AshutoshChenia, HafizahLee, JunSingh, GurpreetCherkaoui-Malki, MustaphaLee, Jun SikSingh, KamalendraChern, Ming-KaiLee, JunsooSingh, NishaCherniack, E. PaulLee, Kin Wah TerenceSinha, Birandra K.Chetcuti, Michael J.Lee, Kwang HoSinicropi, AdalgisaCheung, Randy Chi FaiLee, Lin-wenSiniscalco, DarioCheung, Wing-HoiLee, Meng-JenSiodłak, DawidChew, Eng-HuiLee, Mi KyeongSirusta, SilviaChi, Chun Y.Lee, Min WonSisto, MargheritaChi, YunLee, MinaSiwek, AgataChia, Win-LongLee, Myung G.Sjogren, KlaraChiang, Cheng-YangLee, Sang KookSkalicka-Woźniak, KrystynaChiang, Hsiu-MeiLee, Sang KyuSkaltsa, Helen D.Chiang, Long Y.Lee, Sang-hanSkaltsounis, Alexios-LeandrosChiang, Shen-ShihLee, Seong-WookSkardal, AleksanderChiang, Tai-anLee, Seung HeeSkellam, ElizabethChiang, Wen-DeeLee, Shyi-LongSkenderi, Katerina P.Chiarelli, LaurentLee, So YeongSkovgaard, OleChi-Feng, HungLee, SunwooSlavik, RogerChilmonczyk, ZdzisławLee, Tae SooSławiński, JarosławChimento, AdeleLee, Tai-LinSledz, PawelChin, Young-WonLee, Tse-MinSłoczyńska, KarolinaChinchilla, RafaelLee, TuSmani, YounesChinnici, FabioLee, Tzong-shyuanSmart, LauraChiocchia, GillesLee, Wing-HinSmeds, Annika I.Chiou, Shih-hwaLee, Yong RokSmith, CarolineChiou, Wen-feiLee, Yueh-LunSmith, Charles J.Chisholm, John D.Leeper, Finian J.Smith, Judith A.Chitwood, DavidLegentil, LaurentSmith, Robert A.Chiu, Tien-LungLehnherr, DanSmith, TerryChiu, Tsai-HsinLei, AiwenSmoleński, PiotrChiva-Blanch, GemmaLei, DangyuanSmoliga, JamesChivers, Ian H.Lei, WeiweiSobkowski, MichalChlichlia, KaterinaLeiherer, AndreasSobral, Abilio J. F. N.Cho, ChungheeLeisner, Jørgen J.Socaciu, CarmenCho, Dong-WooLekka, MalgorzataSoengas, RaquelCho, HyunahLemus, JesúsSoeta, TakahikoCho, Man-HoLen, ChristopheSohn, JeongwonCho, NamjoonLenardão, Eder J.Sohtome, YoshihiroCho, Somi KimLenoir, IreneSokolove, JeremyChobot, VladimirLentini, GiovanniSolà Alberich, RosaChoi, Eun-MiLeondaritis, GeorgeSolano, FranciscoChoi, Guang J.Leonetti, FrancescoSolfrizzo, MicheleChoi, HyeonsonLeong, David TaiSolinís, M. ÁngelesChoi, Hyung-KyoonLeoni, AlbertoSolomon, MelaniChoi, Ki ChoonLeonidas, Demetres D.Soloshonok, Vadim A.Choi, Seok KiLephart, Edwin D.Solsona, BenjamínChoi, SeokheunLeporatti, StefanoSone, HidekoChoi, Young HaeLerm, MariaSong, JaewhanChoi, Young HeeLescrinier, EvelineSong, JinhuaChoi, Yung HyunLesyk, RomanSong, JuhaCholewinski, GrzegorzLeung, BernardSong, KenanChoshi, TominariLeung, Chung-HangSong, Kwang HoChou, C. JamesLeung, Elaine Lai-HanSong, MeiChou, Timothy Y.Leung, IvanhoeSong, Sang HoonChoy, Jin-HOLeung, KenSong, Shao-JiangChrissanthopoulos, AthanassiosLevy, ArthurSong, Yang-heonChristensen, Jørn B.Lewinska, AnnaSonsalla, Patricia KChristensen, LarsLewis, KarenSopade, Peter A.Chrobok, A.Lewkowicz, ElizabethSørensen, Jens LauridsChronis, NikosLewkowski, JaroslawSorokin, AlexanderChruszcz, MaksymilianLeyssens, TomSosa, SilvioChrzanowski, ŁukaszLhiaubet, Virginie LyriaSoto, CarmenChu, DafengLi, ChunshunSoukup, GarrettChu, JiaxiangLi, ChunxiSoukup, Sebastian T.Chu, ShidongLi, DongmeiSoulère, LaurentChua, Mei-SzeLi, DongyeSousa, EmíliaChuang, Li-yehLi, GuijieSouza-Smith, FlaviaChuang, Show-meiLi, HouJinSpangenberg, BerndChuang, Ta-hsienLi, HuabinSpano, GiuseppeChueh, Pin JuLi, HuiSparr, ChristofChulvi, KatherineLi, JieSpeck, Peter G.Chun, Byung-sooLi, LiSpégel, PeterChung, DonghoonLi, Mei-LingSpingler, BernhardChung, Jong-WookLi, NianguangSpiteller, PeterChung, Sang JeonLi, PengchengSpyrakis, FrancescaChung, SoonkyuLi, PingSreerama, SubramanyaChung, Yoon-SokLi, QingliangSri Vedavyasa Sri, RadhakrishnanChurchill, Mair E. A.Li, RuiSridhar, JayalakshmiChwalbe, Carl H. S.Li, Shao-ShunSrinivas, KeerthiChyau, Charng-CherngLi, ShujunSrivatsan, Eri S.Cichero, ElenaLi, SihengSrivatsan, MalathiCidade, HonorinaLi, TiejunStacchiotti, AlessandraCieśla, ŁukaszLi, Wei-linStahl, FrankCimanga, KanyangaLi, WeirongStaines, K.A.Ciofu, OanaLi, Wen-WuStallforth, PierreCirillo, GiuseppeLi, XianStaneva, GalyaCirrincione, GirolamoLi, Xiang-GuoStanley, JessicaCirvilleri, GabriellaLi, XiaoboStapleton, P. A.Cisnetti, FedericoLi, XiyouStavropoulos, George P.Citores, LuciaLi, YimengStavros, VasiliosCiulli, AlessioLi, YingxiaStec, David E.Ciurli, StefanoLi, YinyinStec, JozefCivra, AndreaLi, YongleStecca, BarbaraClark, Daniel A.Li, YuliangStefanidis, Georgios.Clements, CarolLi, ZhenStehle, Jörg H.Clift, MartinLi, Zhi-MinhgSteindel, MárioCobbold, SimonLi, ZhongjingSteingass, Christof B.Cocetta, GiacomoLi, Xu-WenStella, LorenzoCock, Ian EdwinLiagre, B.Stenstrøm, YngveCoelho, José A.Liakos, Ioannis L.Stephen, Michael RajeshCognasse, FabriceLiang, DongStevenson, PaulCohen, HaimLiang, KangStewart, Alan V.Coley, Helen M.Liang, LiStewart, AlexandreColl, JosepLiang, Po-huangStewart, Cameron R.Collins, MauriceLiang, Shih-ShinStewart, Teneale A.Collins, Stuart G.Liang, Xin-miaoSteyn, FrederikCollis, Gavin E.Liang, YongStiborova, MarieColombo, ClaudioLiang, YuerongSticherling, MichaelColquhoun, Thomas A.Liao, Ai-HoStierle, AndreaColumbano, AmedeoLiao, DaiqingStine, Keith J.Comai, StefanoLiao, Hui-FenStipanovic, ArthurConceição, KatiaLiao, PengStiriba, Salah-EddineConsonni, RobertoLiaw, Chih-ChuangStirpe, FiorenzoConstantino, ValeriaLidon, Fernando CebolaStockdill, Jennifer L.Conti, BarbaraLieberzeit, PeterStockland, Robert A.Conti, PioLiermann, Johannes C.Stojakowska, AnnaContreras, AngelaLiew, ChongweeStone, WilliamContreras, Lucila GarciaLifshitz, EfratStournaras, ChristosContreras, María Del MarLigon, Lee A.Strano, SabrinaCooper, ArthurLill, Markus A.Stratakis, ManolisCorchete, PurificacionLim, CheeStrekowski, LucjanCorcione, Carola EspositoLim, Dong WookStreubel, RainerCordoba-Diaz, D.Lim, Dong-KwonStrube, JochenCorella, DoloresLim, YoonghoStruga, MartaCorke, HaroldLima, Raquel T.Stuchebrukhov, AlexeiCormode, David P.Lima-Filho, José V. M.Studt, LenaCornaglia, M.Lin, Chih-LiStüger, HaraldCorral, InésLin, Dr. Henry C.Su, Chun-LiCorrea-Basurto, JoseLin, Hai-shuSu, ChunmingCorreia Soeiro, Maria De NazaréLin, HuiSu, HuanxingCorreia-de-Sá, PauloLin, Hung-YinSu, Jui-HsinCortes Corberán, VicenteLin, Kuo-ShyanSu, Xiao QCosta, Elisabete V.Lin, Liang-TzungSu, ZhiqiangCosta, Maria Do Céu Gonçalves DaLin, Li-GenSu, Zi-RenCosta, RosariaLin, ShangchaoSubramaniam, DharmalingamCosta Pessela João, BenevidesLin, Shyh-HsiangSubramaniam, PrasadCostantini, MariaLinert, WolfgangSucheck, StevenCostantino, FerdinandoLing, ChenSuckling, Colin J.Costantino, ValeriaLing, Yong-ChienSuga, HiroakiCostas, MiquelLink, WolfgangSuga, SeijiCouce, María D.Linz, John ESugahara, TakuyaCouch, RobinLiou, Chian-JiunSugai, TakeshiCournia, ZoeLiou, Ying-mingSugimoto, KenjiCowan, TinaLisboa, PatríciaSugiura, YoshimasaCox, JaneLisi, LucianaSuh, Hyung JooCox, Russell J.Litinas, Konstantinos E.Suib, Steven L.Cozzolino, DanielLittle, R. DanSuklje, KatjaCrabtree, Robert H.Liu, BaomingSukocheva, OlgaCranfield, CharlesLiu, BifengSulman, EstherCrean, DanielLiu, Cheuk LunSun, Dr. GraceCrecchio, CarmineLiu, ChongSun, GuohuiCrespo, MargaritaLiu, Chun-yanSun, JianCrinò, PaolaLiu, DajiangSun, LitaoCrispin, Matthew C.Liu, DelongSun, PeilongCrisponi, GuidoLiu, FeiSun, ShiCruse, RichardLiu, GuijianSun, WenjieCsámpai, AntalLiu, Hai-yangSun, ZhonghuaCsiszár, AgnesLiu, HongSunagar, KartikCsuk, ReneLiu, HongbingSunatsuki, YukinariCuculovic, MilosLiu, Hui-lingSunavala-Dossabhoy, GulshanCuellar, MauricioLiu, JinSung, Mi JeongCui, ZiningLiu, Jin-GangSung, Ping-JyunCullis, ChristopherLiu, Jun-JenSunseri, FrancescoCunha, MárioLiu, LeiSurup, FrankCurti, ClaudioLiu, LiangSutton, TroyCustódio, LuísaLiu, LiangliangSuzuka, ToshimasaCutignano, AdeleLiu, PengSvedberg, Marie M.Cutrignelli, AnnalisaLiu, RenyuSweetman, CrystalCzechowski, TomaszLiu, Shih-JungSwindle-Reilly, Katelyn E.Czerwinska, MonikaLiu, Shing-HwaSwinney, David C.Da Costa, Gonçalo GamboaLiu, Shiuh-TzungSyed, ViqarDa Motta, M.Liu, XiaojingSykes, MelissaDa Silva, Edson RobertoLiu, XiaoxiSzekely, GyorgyDa Silva, Felipe M. A.Liu, XinSzilvási, TiborD’abrosca, BrigidaLiu, YanfangSzöllősi, DanielDabrowski, JanuszLiu, Ying-LingSzpilman, Alex M.D’accolti, LuciaLiu, YonghongSztanke, MałgorzataDacheux, Jean-LouisLiu, YongqiangSzumny, AntoniDai, HaofuLiu, Yuan-ShuaiSzunerits, SabineDai, YuminLiu, YueTabanca, NurhayatDai, YunLiu, YujunTabata, YasuhikoDai, ZhanwuLiu, Zhi LongTachibana, ShinjiroDaina, AntoineLiu, JialeiTadić, VanjaDal Monte, MassimoLizcano De Vega, José MiguelTaherzadeh, MohammadDalhoff, AxelLlona-Minguez, SabinTahir, Prof. NawazDall´Acqua, StefanoLloyd-Williams, PaulTai, Cheng-JengDaly, SethLo, Kenneth Kam-WingTaillefumier, C.Da’na, EnshirahLo, Lee-Chiang LoTaira, JunseiDang, Jing-ShuangLo Scalzo, RobertoTajima, TakahisaDaniels-Wells, Tracy R.Locatelli, MarcelloTak, Jun-HyungDanneels, Ellen L.Loh, Xian JunTakagaki, AtsushiDar, Mohammad IbrahimLoizzo, Monica RosaTakahama, UmeoDardonville, ChristopheLok, Chun NamTakahashi, DaisukeDas, DeepankarLombó, FelipeTakanami, ToshikatsuDas, ViswanathLongo, PasqualeTakano, KatsuraDasari, RameshLongo, VincenzoTakebe, NaokoDasgupta, PiyaliLongstaffe, James G.Takei, TakashiDasgupta, SayaniLoos, KatjaTakenaka, ShinjiDaugaard, Anders EgedeLopez, Dolores E.Takeshita, FumitakaD’Auria, JohnLopez, LuisTakita, RyoD’Auria, Maria ValeriaLopez, VictorTakizawa, ShinobuD’Auria, MaurizioLópez, José ManuelTalbert, Joey N.Dauwe, RebeccaLópez, María L.Talele, Tanaji TDaverey, AmitaLópez-Abán, JulioTam, KinDavies, Gemma-LouiseLópez-Bucio, J.Tamanoi, FuyuhikoDavis, BurtLopez-Garzon, RafaelTamassia, NicolaDavis, RohanLopez-Lluch, GuillermoTame, JeremyDawadi, SurendraLoppi, StefanoTamura, OsamuDe Aberasturi, Dorleta JiménezLord, MeganTan, Cai-PingDe Almeida, Martha Elisa FerreiraLorenzi, VanninaTan, DunxianDe Almeida Coelho De Sousa, Maria JoãoLoris, RemyTan, JianwenDe Aza, Piedad N.Loschen, ChristophTanahashi, TakaoDe Beer, DaleneLou, SongTanaka, Aparecida SadaeDe Caro, VivianaLoveridge, JoelTanaka, NaokiDe Castro, SolangeLowary, ToddTanaka, ShigenoriDe Corato, UgoLu, Chung-kuangTanaka, TakayukiDe Dea Lindner, JulianoLu, FaChuangTang, You-ZhiDe Falco, EnricaLu, JunTang, YuDe Feo, VincenzoLu, Jyh-FengTang, Yu-PingDe Figueiredo, MarciaLu, Mei-ChinTanida, MamoruDe Freitas, VictorLu, MingTaniguchi, NobukazuDe Freitas Gauze, GiseleLu, Qi-longTanimoto, HirokiDe Geest, BartLu, ShuoTantillo, DeanDe Graaf, CoenLu, YongenTao, YuanqiDe Juan, AnnaLucas, David M.Tappia, Paramjit S.De Kimpe, NLucini, LuigiTarantino, GiovanniDe La Hoz, AntonioLudwiczuk, AgnieszkaTarasova, Nadya I.De La Monte, SuzanneLugemwa, Fulgentius NelsonTarkowska, DanuseDe La Torre, Maria Del CarmenLui, Edmund M.K.Tarr, D EllenDe Luca, StefaniaLuis, Santiago V.Tava, AldoDe Melo Barbosa Dekker, AneliŁukasik, RafałTavano, LorenaDe Mendonça, Dina I. M. D.Lukyanov, Pavel A.Tavares Carvalho, José CarlosDe Menezes, Irwin R. A.Luna, A.s.Tavazzi, BarbaraDe Pauw, EdwinLuna, Jonny E. DuqueTavío, Maria Del MarDe Rijke, EvaLundén, KarlTawata, ShinkichiDe Vendittis, EmmanueleLung, Ming-YeouTaylor, James E.De Vries, MattanjahLuo, JianTempesta, MariaDeda, Massimo LaLuo, Qi CongTerán, CarmenDehaen, WimLuo, Shi-ZhongTerner, JamesDekanski, DraganaLuo, YinggangTerzyk, Artur P.Dekker, FrankLupiáñez, José A.Teschke, RolfDel Bonis-O’Donnell, Jackson TravisLuque, F. JavierTessore, FrancescaDel Mercato, LorettaLustri, WiltonTeufel, RobinDel Pozo Losada, CarlosLyons, RussellTewari-Singh, NeeraDelattre, CédricMa, Choong JeTezuka, YasuhiroDelgadillo, Adriana San MiguelMa, GuipingThakur, ChitraDella Ca, NicolaMa, XiaoqianThamattoor, Dasan M.DellaGreca, MarinaMa, YongThiéry, ValérieDelpino, AntoniMa, YuemingThiriet, MarcDemchenko, AlexeiMaas, MichaelThirunavukkarasu, MaheshDemirci, Prof. Dr. FatihMaccallini, CristinaThomas, EricDen Hertog, JeroenMacchi, BeatriceThomas, Sajesh P.Denat, FranckMacedo, Maria LígiaThomas, SamuelDeng, JianjunMachetti, FabrizioThomas, ShaneDeng, JunMaciejewska, DorotaThomas, Stephen P.Deng, Lih-WenMaciel, PatríciaThomas, T. J.Dentino, Andrew R.Mackerell, Alexander D.Thomas, Walter G.Derat, EtienneMacknight, Richard C.Thöming, GundaDesimone, Martin FedericoMackowski, SebastianThomsen, Allan RandrupDessy, ChantalMaçôas, ErmelindaThoo-Lin, Paul KongDeurwaerdère, PhilippeMadar, ZechariaThorwart, MichaelDevamani, TituMaddau, L.Thota, SreekanthDevriendt, BertMadesis, PanagiotisThounaojam, MenakaDey, PriyankarMadsen, Andreas S.Thunders, MichelleDhakshinamoorthy, AmarajothiMaeda, HayatoTidwell, ThomasDhanasekaran, Danny N.Maeda, KatsuhiroTietjen, IanDi Luca, MariagraziaMaestro, MiguelTimperley, Christopher M.Di Maro, AntimoMafra, IsabelTing, RichardDi Martino, PieraMagalhães, Júlia M. C. S.Ting, SimonDi Santo, RobertoMagata, YasuhiroTiritan, Maria ElizabethDi Serio, MartinoMaggi, FilippoTitinchi, SalamDi Somma, IlariaMagnaldo, ThierryTitorenko, Vladimir I.Diana, PatriziaMaguire, AnitaTiziano, TuccinardiDiana, PatriziaMaher, PamelaTognetti, VincentDíaz, José FernandoMahmudov, KamranTojo, EmiliaDiederich, MarcMaicas, SergiTokuyama, ShogoDíez, DavidMaier, CameliaTolosa, JosefaDijkhuizen, LubbertMaier, Jeanette A.m.Tomalia, DonaldDikalov, SergeyMaier, PatrickTomé, Augusto C.Ding, ChunbangMaione, FrancescoTomi, FélixDing, JunqiaoMaisch, TimTomicic, MajaDinh, Dzung H.Majumdar, SusrutaTomitaka, AsahiDiniz, CarmenMak, King Lun KingstonTommasi, SaraDipali, SharmaMäkelä, Jyrki M.Tommonaro, GiuseppinaDixon, David A.Makihira, SeichoTomoda, HiroshiDjabali, KarimaMäkinen, SariTomofuji, TakaakiDjordjevic, SnezanaMakishima, MakotoTonelli, MicheleDmitriev, RuslanMakosza, MieczyslawToohey, JohnDobrev, DobromirMalachová, AlexandraTooyama, IkuoDolzhenko, Anton V.Malaguarnera, MicheleTop, SidenDomashevskiy, Artem V.Malawska, BarbaraTormo, José RubénDomcke, WolfgangMalda, JosTörök, MariannaDominguez, HerminiaMalde, AlpeshToropov, Andrey A.Domínguez-Álvarez, EnriqueMaldini, MariateresaTorrens, FranciscoDonalisio, ManuelaMale, David K.Torrents, EduardDong, F.Malheiro, RicardoTorricelli, RenzoDong, MingdongMalkoch, MichaelTouchard, AxelDongari-Bagtzoglou, AnnaMallipeddi, Prema LathaToy, RandallDonno, DarioMaloney, MarkToydemir, GamzeDorta, RetoMalucelli, GiulioToyoda, AtsushiDos Santos, Jean LeandroMalumbres, MarcosTraba, C.Dosio, FrancoMamane, VictorTrabace, LuigiaDou, Q. PingMambu, LengoTrabolsi, AliDouroumis, DionysiosMan, DulaTresini, MariaDovinova, ImaManabe, ShinoTrincone, AntonioDowden, JamesManca, GabrieleTrindade, Reginaldo Almeida daDrabowicz, JozefManca, Maria LetiziaTringali, CorradoDrabowicz, JózefMancheño, JoséTripathi, Siddharth KaushalDragonetti, ClaudiaMancheño, Olga GarciaTripodi, GianlucaDraijer, RichardMancini, InesTripp, Carl P.Drasar, PavelManda, Vamshi K.Trippier, Paul C.Du, HaifengMandal, DebashisTrivedi, Drupad K.Du, ShushanMañes, JordiTrombetta, DomenicoDuan, JinaoMang, Thomas S.Trombini, ClaudioDuan, XiaohuiMangelinckx, SvenTruchado, PilarDuarte, Ana Rita C.Mannino, SaverioTrujillo Rodríguez, María JoséDuarte, FátimaMano, JunichiTruong Phuoc, NghiaDuarte Campos, Daniela FilipaMano, YujiTrzeciak, AnnaDuba, KurabachewManos, ManolisTsai, Cheng-FangDubey, RaminManrique, CamilaTsai, Chia-FangDubois, JoëlleMansfield, Anna KatharineTsai, Chia-WenDubois, MarcMantovani, GiuseppeTsai, Fuu-JenDucki, SylivieManzo, EmilianoTsai, I-LinDudás, JózsefMao, ChuanbinTsai, SherylDudding, TravisMao, Zong-WanTsai, Tsung-YuDüfer, MartinaMarakos, PanagiotisTsang, Siu WaiDuggan, BrendanMarc, DanielTsantili-Kakoulidou, AnnaDughera, StefanoMarcelli, AugustoTsao, Chia-WenDugo, GiacomoMarchand, PascalTsay, Hien-YieDuh, Chang-YihMarchiani, SaraTseng, Chih-HuaDuke, Stephen O.Marchi-Delapierre, CarolineTseng, Wei-LungDumas, FrançoiseMarco-Contelles, JoséTsim, KarlDumont, EliseMarcos, MercedesTsipis, Athanassios C.Dumur, FrédéricMarcotullio, CarlaTsoleridis, Constantinos A.Dunietz, Barry D.Marczak , JacekTsopmo, ApollinaireDuong, Hai M.Maresca, MarcTsotsis, Theodore T.Dupont, Daniel M.Marestin, CatherineTsoungas, Petros G.Duran Lobato, MatildeMárialigeti, KárolyTsugawa, HiroshiDurand, Jean OlivierMariappan, KadarkaraisamyTsui, Stephen Kwok-WingDurazzo, AlessandraMarin, D.E.Tsuji, HayatoDusselier, MichielMarin, Jose J. G.Tsuji, YasuhideDust, Julian M.Marini, FrancescaTsukamoto, MasakiDuval, Raphael E.Marinkovic, NebojsaTsukiyama-Kohara, KyokoDzantiev, BorisMarinozzi, MauraTsurkan, Mikhail V.Dziadek, JarosławMarkopoulos, JohnTsutsui, MakusuDziedzic, ArkadiuszMarkopoulou, OlgaTuão Gava, Carlos AlbertoDziubla, ThomasMarkossian, SarineTuccinardi, TizianoDzyuba, SergeiMarkovitsi, DimitraTufarelli, VincenzoEasmon, JohnnyMaroja, Luana S.Tulkens, PaulEbrahim, WeaamMarques, ClaudiaTulla Puche, JuditEchevarria, AureaMarquetand, PhilippTullius Scotti, MarcusEcker, Gerhard F.Marra, AlbertoTung, Bui ThanhEdokpayi, JoshuaMarrocchi, AssuntaTuononen, Heikki M.Efferth, ThomasMarschner, ChristophTurrini, EleonoraEfimia, HatzidimitriouMarsh, LorraineTyagi, PradeepEguchi, YutakaMarsura, AlainTyler, BettyEiden, Lee E.Marti, GuillaumeTyndall, JoelEjima, HirotakaMartin, JessicaTzakos, Andreas G.Ekuni, DaisukeMartin, JuliaUbiali, DanielaEl-Aneed, AnasMartin, MathewUbukata, TakashiEldeeb, MohamedMartín, Juan F.Uccello-Barretta, GloriaEleftherianos, IoannisMartín-Banderas, LucíaUddin, ZakirElisabetta, CasalinoMartinelli, AdrianoUehara, TomoyaEl-Kadi, Ayman OSMartínez, AlbertoUeno, TakafumiEl-Safty, Sherif A.Martínez-Palou, RafaelUji-I, HiroshiEl-Seedi, Hesham R.Martin-Ramos, PabloUkic, SimeElsinghorst, Paul W.Martins, SoniaUlatowski, LynnEmanuelsson, EmmaMartin-Sampedro, RaquelUlisse, SalvatoreEmmott, EdwardMartra, GianmarioUllah, AmanEmpting, MartinMaruyama, KeiUlrich, CraigEngels, JoachimMarverti, GaetanoUlrich, JoachimEngle, Keary M.Marzaro, GiovanniUno, TomohideEparvier, VéroniqueMarzocco, StefaniaUnrau, PeterEpifano, FrancescoMasaoka, ShigeyukiUrbani, MaxenceEpstein, AlanMase, NobuyukiUrbanska, Ewa M.Erdélyi, MátéMasek, AnnaUrbieta, María TaberneroEriksson, Leif A.Maserti, Bianca ElenaUrra, José MiguelEritja, RamonMassa, AntonioUsami, YoshihideErtl, PeterMassaro, MarikaUsui, KazuteruErxleben, AndreaMasterson, DouglasUsui, KenjiEsatbeyoglu, TubaMastrangelo, Anna M.Usui, TakeoEscalante García, JaimeMasu, HyumaUsuki, YoshinosukeEscudier, Jean-MarcMasurier, NicolasUtsumi, ShigenoriEscuredo, OlgaMata, I.Vachali, PreejithEspinosa Aguirre, JavierMatar, Samir F.Vajda, SandorEspinosa-Diez, CristinaMateos-Timoneda, Miguel A.Vakkilainen, EsaEsposito, SergioMaterić, DušanValcheva-Kuzmanova, StefkaEsteban, Maria AngelesMaterska, MałgorzataValdes, EsperanzaEstevez, Ramón J.Mathesius, UlrikeVale, NunoEstévez-Braun, AnaMathieu, DidierVale, PauloEstevinho, LeticiaMatiadis, DimitrisValencia, GregorioEtchenique, RobertoMatin, AbdulValentao, PatríciaEto, BrunoMatosiuk, DariuszValente, SergioEuaggelos, GikasMatsubara, KeiichiValentova, KaterinaEvanno, LaurentMatsuda, HisashiValero, ManuelEvans, PaulMatsugo, SeiichiValgimigli, LucaEverts, BartMatsui, KenjiValiyaveettil, SureshExarchou, VassilikiMatsui, MasakiValles, Soraya L.Fábián, LászlóMatsui, NobuakiVan Calenbergh, SergeFabiano-Tixier, Anne-SylvieMatsui, Toshirovan den Broek, LambertusFabre, NicolasMatsui-Yuasa, IsaoVan Den Ende, WimFaccio, GretaMatsumoto, KazuyaVan Der Stelt, MarioFaggio, CaterinaMatsumoto, YasuhikoVan Dyke, KnoxFakis, MihalisMatsumura, KiyoshiVan Ginkel, PaulFamiani, F.Matsumura, ShuichiVan Griensven, Leo J. L. DFan, HongMatsuno, KojiVan Lanen, StevenFan, QinghuaMatsuoka, Shin-ichiVan Tassel, PaulFanali, ChiaraMatsuzaki, YasushiVan Waardenburg, Robert C. A. M.Fañanas, Francisco J.Mattivi, FulvioVanamala, Jairam K. P.Fanfrlík, JindřichMattoo, AutarVanderlei, Maria de FátimaFang, BaiMaurel, Marie-christineVanelle, PatriceFang, MarongMavila, SudheendranVangala, VenuFanizzi, Francesco P.Mavromoustakos, ThomasVankayala, RavirajFanning, Kent J.Mayence, AnnieVarani, LucaFant, Jeremie B.Mayhan, William G.Varela-López, AlfonsoFaraji, ShirinMazerska, ZofiaVarela-Ramirez, ArmandoFarantos, Stavros C.Mazzeo, MarcoVaresano, AlessioFaria, Jorge M.S.Mazzola, EugeneVargas, Rubem M. F.Farinati, FabioMazzone, GloriaVarvounis, GeorgeFarris, StefanoMazzoni, LucaVasilyev, AleksanderFasani, ElisaMbosso, Emmanuel Jean TeinkelaVaughan, Roger A.Faure, ChrystelMcardle, PatrickVávrová, KateřinaFaure, SébastienMcCallum, Jason L.Vavvas, Demetrios G.Faustino, Maria AmparoMcCann, Mark J.Vazquez-Martínez, José AlfredoFávero, Priscila PereiraMccarley, Robin L.Vegvari, AkosFedorova, MariaMcCormick, Alon V.Veiga Jr, Valdir FlorencioFedrizzi, BrunoMcFeeters, Robert L.Veleeparambil, ManojFeliers, DenisMcilroy, David N.Velez, ZéliaFelinger, AttilaMcIntosh, Marla S.Velkov, TonyFelipe Farias, DaviMcLemore, GabrielleVenditti, AlessandroFelpin, François-xavierMcNamara, BruceVennerstrom, JonathanFeng, QuanyouMcPhail, KerryVenus, JoachimFeng, WeishengMcRae, Jacqui M.Vepsäläinen, JoukoFeng, Wen-haiMeagher, LaurenceVergadi, EleniFeng, YangboMeagher, RobertVergara, DanieleFeng, YibinMebs, DietrichVerma, DeeptakFeng, Yong-LaiMedana, ClaudioVerma, MadanFeng, YuMedeiros, RodrigoVerma, RanjitFennell, Timothy R.Medici, SerenellaVerma, SannyFeo, FrancescoMedvidović-Kosanović, MartinaVernekar, Sanjeev KumarFernandes, EduardaMehbub, Mohammad FerdousVerri Jr., Waldiceu A.Fernandes, PedroMehta, Akul Y.Vertuani, SilviaFernandes, Susana C.M.Mei, XuefengVervoort, JacquesFernandes-Ferreira, ManuelMeille, ValérieVetter, Stefan W.Fernandez, EstherMeinke, MartinaVetvicka, VaclavFernández, Francisco J.Melino, SoniaViapiana, AgnieszkaFernández, InmaculadaMelo, AndréVidal, JoëlleFernández, José J.Melone, LucioVideira, ArnaldoFernández, RosarioMelzig, MatthiasVieira, MónicaFernandez Megia, EduardoMemarzadeh, KavehVignolle, JoanFernandez-Garcia, MartaMena-Rejon, Gonzalo J.Vilanova, ManuelFernandez-Lafuente, RobertoMenendez Ramos, José CarlosVilanova, NeusFernández-Recio, JuanMenet, Marie ClaudeVilela, AliceFeron, KrishnaMenezes, JoséVillalobos, CarlosFerrari, ErikaMeng, HuanVillalonga, ReynaldoFerraris, Dana VMenkhorst, EllenVillani, ClaudioFerraz, RicardoMenna, MarialuisaVillemagne, Victor L.Ferré-D’Amaré, Adrian R.Menut, PaulVilloutreix, BrunoFerrero, FrancoMercier-Bonin, MurielVinale, FrancescoFerretti, ValeriaMercolini, LauraViñas, MiguelFigadère, BrunoMerfort, IrmgardVincenzo, LattanzioFigueira, EtelvinaMerino, PedroVinogradov, EvgueniiFigueiral, Maria HelenaMerlini, LucioVirjamo, VirpiFigueiredo, Ana CristinaMerlino, AntonelloVisa, MariaFiguerola, AlbertMerlo, Aloir AntonioViso, AlmaFilarowski, AleksanderMero, AnnaVistoli, GiulioFilipovic, Milos R.Mesías García, MartaVitalini, SaraFilosa, RosannaMesseguer, AngelVitillo, Jenny G.Fiocco, DanielaMessina, FedericaVitorino, RuiFisar, ZdenekMestres, Adrià GilVittadini, AndreaFisher, Jed F.Métro, Thomas-XavierViuda-Martos, ManuelFissell, WilliamMetzinger, LaurentVivo, PaolaFitó, MontserratMeulia, TeaVlase, LaurianFitton, HelenMewis, IngaVo, Minh D.Fleischer, IvanaMeyer, Hans-PeterVolety, Aswani K.Fleming, IngridMeyers, Marvin J.Volpe, MariaFletcher, JamesMező, GáborVon Der Mark, KlausFlood, AmarMezzenga, RaffaeleVon Wright, AtteFloreancig, Paul E.Mhd Jalil, Abbe MaleykiVosteen, IlkaFlorian, PitterlMichalcova, LenkaVougas, KostasFochi, MariafrancescaMichiels, CarineVougogiannopoulou, KonstantinaFons, FrançoiseMicura, RonaldVoutquenne, LaurenceFoo, EloiseMidgley, AdamVu, Chi B.Forlani, GiuseppeMiecznikowski, K.Vuong, Quan V.Forsyth, Christopher B.Miele, Adriana E.Vuong, Quan VanFort, FrancescaMigone, AldoWada, YujiFortes, ZuleicaMiguel, GraçaWadsak, WolfgangFortier, SkyeMíguez, HernánWagner, AnikaFossa, PaolaMija, AliceWagner, GabrieleFossen, TorgilsMikata, YujiWahab, M. FarooqFossum, MagdalenaMiki, YasuhiroWaheed, AbdulFoster, Brian CMiklossy, GabriellaWahyudi, HendraFosu-Mensah, NellyMikołajczyk-Bator, KatarzynaWakabayashi, HirokiFouassier, LauraMikulski, DamianWaksmundzka-Hajnos, MonikaFoubelo, FranciscoMilanesio, MarcoWaldmeier, FelixFoulongne-Oriol, MarieMilcarek, ChristineWalker, JessicaFourmy, DanielMilella, LuigiWalker III, William B.Fox, GlennMilelli, AndreaWallis, RussellFraga, Marco A.Miller, JohnWalls, ZacFraile, AlbertoMiller, Marshall G.Walsby, Charles J.Fraile, José M.Millet, OscarWalser, Tonya C.Franca, Eduardo FMillo, EnricoWaltenberger, BirgitFranceschinis, E.Mimaki, YoshihiroWan, JianboFrancesco, MasoeroMimica-Dukic, NedaWan, Xiao-ChunFrancioso, AntonioMinbiole, Kevin P. C.Wan, XuehuaFrank, SašaMindt, Thomas L.Wang, Chang-hongFranzke, Claus-WernerMinguillón, CristinaWang, Chao-MinFreire, FélixMinutolo, FilippoWang, Chi ChiuFreitas, MarisaMiranda, CristobalWang, Chin-KunFribley, Andrew M.Miranda, KatrinaWang, Chrong-ReenFroeyen, MatheusMiron, Richard J.Wang, CundeFrolova, LiliyaMirzayans, RazmikWang, DavidFrosini, MariaMisaki, TomonoriWang, DongDongFruhmann, PhilippMishra, VandanaWang, Gou-JenFu, GangMitakou, SofiaWang, HaiqiaoFu, JunjieMitchell, JaneWang, I-JongFu, QiangMiura, TsuyoshiWang, JeffreyFuchs, HendrikMiyabe, HidetoWang, Jeh-jengFudala, RafalMiyamoto, KojiWang, JinFujihara, TetsuakiMiyamoto, ManabuWang, JunjianFujii, KotaroMiyazaki, ToshikiWang, LiliFujimori, KoMiyazato, HironariWang, LipingFujimoto, YukariMizuno, Cassia SWang, MichaelFujita, MasahiroMizuno, MasashiWang, PengFujita, MikakoMizushige, TakafumiWang, PiwenFujiwara, RyoichiMizutani, OsamuWang, QuanFukuda, TakeshiMladěnka, PřemyslWang, QunFukushima, AtsushiMlcek, JiriWang, Robert Y. -L.Fukushima, MasamiMłochowski, JacekWang, SanwuFuller, Amelia A.Mlsna, ToddWang, Sheng-yangG. Lago, João HenriqueMoal, IainWang, WeiGadotti, Vinicius MMohamed, RamdaniWang, Wei-LungGai, FrancescoMohanam, SanjeevaWang, WentianGalanis, AlexMohanta, TapanWang, XiangkeGalan-Vidal, Carlos AndresMok, Daniel Kam-WahWang, Xiao-BingGaldiero, MassimilianoMolasiotis, AthanasiosWang, XiaohongGaleazzi, RobertaMolinari, AlessandraWang, XiaosongGales, LuísMolinari, FrancescoWang, XiaowuGallagher, John F.Molinski, TadeuszWang, XinGallego, SergiMollahosseini, MehdiWang, XinkunGallo, AntonioMöller, HeikoWang, Xin-LuanGallo, CarmelaMollica, AdrianoWang, XuGallo, GiuseppeMollica, GiuliaWang, Xue-SongGalvez, JulioMolnar, FerdinandWang, YiGalvez, Maria ElenaMomiyama, NorieWang, YifeiGan, Chee-YuenMonari, AntonioWang, YingGan, RenyouMondal, DebasisWang, YonghengGanesan, A.Moneim, Ahmed E. AbdelWang, Yun-MingGanguly, AnutoshMonflier, EricWang, ZhengtaoGao, BaoxiangMonge, DavidWang, ZhiweiGao, JieMonk, JenniferWang, ZuankaiGao, JinmingMonopoli, AntonioWangchuk, PhurpaGao, Li QianMonsees, ThomasWansi, Jean DuplexGao, SongMontalbano, AlessandraWard, Sara JaneGao, XueMontazeri Aliabadi, HamidrezaWaseem, RazaGaraj, VladimírMonteil-Rivera, FannyWaser, MarioGaravelli, MarcoMonteiro, Michael J.Wąsik, Tomasz J.Garbers, ChristophMontenegro, LuciaWatanabe, MasatoshiGarcia, DiegoMoon, Sang-HoWatanabe, RyuichiGarcia, JordiMoraca, FedericaWatanabe, TatsuoGarcía, TaniaMoraes, Valéria R SWatkins, A. NealGarcía Csákÿ, AurelioMorales, DavidWatkins, G. LeonardGarcía-Antón, JordiMorales, JoseWaxman, JoshuaGarcia-Gonzalez, CarlosMorata, AntonioWeber, John TGarcía-Ortega, LucíaMoreira, Irina S.Wehmschulte, Rudolf J.Garcia-Sosa, Alfonso T.Moreira, Maria TeresaWei, Da-HuaGarcía-Verdugo, EduardoMoreira, Paula I.Wei, Guor-JienGarcía-Villalón, Angel LuisMoreira Borges, RicardoWei, HuiGard, PaulMoreno, DiegoWei, QufuGardossi, LuciaMoreno, J. J.Wei, WuGarg, AkhilMorikawa, AtsushiWeinhold, FrankGarg, UttamMorikawa, ToshioWeinstein, Julia A.Garneau-Tsodikova, SylvieMoriwaki, HiroshiWeising, KurtGarrido, Daniel ValeroMoriya, TakahiroWelch, Kevin D.Garrigos Selva, Maria del CarmenMoro, StefanoWelker, Mark E.Garrote, GilMorris, Gordon A.Welte, Cornelia U.Garson, MaryMorris, James C.Wendel, Graciela HaydéeGärtner, PeterMorse, StephenWeng, AlexanderGasbarri, CarlaMoschos, Sterghios A.Weng, Ching-FengGaspar, DiazMostofian, BarmakWeng, DuanGaspar, KrisztianMota, ManuelWeng, Meng-ShihGasparini, PierliugiMotoyama, KeiichiWeng, QunhongGasparri, FabioMotte, LaurenceWertz, Philip W.Gasparrini, MassimilianoMotz, GünterWesolowski, MarekGasser, ChristophMoudgil, Kamal D.West, CarolineGates, WillMourtzinos, IoannisWest, RobertGautier, ArnaudMoussa, FathiWestcott, Stephen A.Gavara, RafaelMoyano, AlbertWeston, LeslieGavicho Uarrota, VirgilioMoyano, LourdesWeston, Paul A.Gavira, José A.Mózsik, GyulaWestwell, AndrewGazerani, ParisaMphahlele, Malose JackWestwood, Nicholas J.Ge, GuangluMuccioli, Giulio G.White, Mark G.Ge, XiaodongMueller, Geoffrey A.Whitford, Paul CGe, XiaoweiMuhle-Goll, ClaudiaWiczk, WiesławGeisler, SarahMühlenhoff, UlrichWiczkowski, WieslawGeletii, Yurii V.Mukherjee, JogeshwarWiesner, MelanieGelmi, Maria LuisaMukherjee, SubhenduWietrzyk, JoannaGendaszewska-Darmach, EdytaMukudai, YoshikiWilking, JanetGenta-Jouve, GrégoryMuller, M.Wilkinson, BrendanGeorge, Riham F.Muller, UlrichWilkinson, J. M.Georgiadi, AnastasiaMüller, ChristophWilkinson, Mark D.Georgiev, VasilMüller, KarstenWilliams, GavinGeppetti, PierangeloMüller, WernerWilliams, Noelle S.Gerber, DoronMulloy, BarbaraWilliams, Robert J.Gerke, JörgMulo, PaulaWilliams, VanceGermain, MarcMun, JiyoungWilliamson, MikeGermani, RaimondoMunoz, MacarenaWillis, Michael C.Geronikaki, AthinaMurai, JunkoWilson, Brenda AnneGerothanassis, Ioannis P.Murai, MasahitoWilson, David B.Ghadwal, Rajendra S.Murali, D.Winckler, ThomasGhanotakis, Demetrios F.Muranaka, AtsuyaWindle, Henry J.Ghavaminejad, AminMurkovic, MichaelWink, MichaelGhignone, StefanoMurray, Deirdre M.Winkler, JohannesGiamperi, LauraMurthi, PadmaWinter, HeikoGiampieri, FrancescaMurugaiyan, JayaseelanWirth, ThomasGiampietro, LetiziaMurugan, N. ArulWisconsin, BloodcenterGianferrara, TeresaMusch, MarkWitcher, MichaelGiannoni, PatriziaMusiol, RobertWitczak, ZbigniewGiaouris, EfstathiosMusso, LoanaWitkowski, StanisławGil, AnaMustafa, ArwaWoelwer-Rieck, UrsulaGilabert-Oriol, RogerMutus, BulentWohl, Christopher J.Giles, GregoryMykhaylyk, OlgaWohlrab, SebastianGillinham, DennisMyllykallio, HannuWojaczyńska, ElżbietaGillman, JasonNa, Dong HeeWojdyło, AnetaGilmer, John F.Nachtigallová, DanaWölfle, UteGilson, MichaelNagai, TakayukiWong, Chung F.Giménez, RaquelNagai, YoshinoriWong, Fung-FuhGindensperger, EtienneNagaoka, SatoshiWong, Ka-LeungGiordano, AntonioNagasawa, KazuoWong, Man-SauGiotta, LiviaNagata, KojiWong, Sze ChoongGiovannoni, Maria P.Nagata, ToshiWong, Vincent Kam WaiGiovarelli, MirellaNagatomo, ShigenoriWood, BaydenGirardon, Jean-SébastienNagulapalli Venkata, KalyanWoodman, OwenGiraudeau, PatrickNagy, PeterWoodward, Robert T.Girbés, TomásNaha, Pratap C.Woon, EstherGirlando, AlbertoNair, VimalWoźniak, DorotaGirousi, StellaNakagawa-Goto, KyokoWrenger, CarstenGitzendanner, MattNakamura, ItaruWright, Colin W.Giuntini, FrancescaNakamura, KozoWu, BingGnavi, GiorgioNakamura, NorikoWu, Bin-NanGohda, EiichiNakanishi, IkuoWu, Chi-ReiGoins, BethNakano, KojiWu, Chung-ChihGoławska, SylwiaNakashima, SouichiWu, Ho-ShingGold, BrianNakatani, YoshihikoWu, HuaGoldsmith, Chloe D.Nalda-Molina, RicardoWu, HuaizhuGolmohamadi, AmirNam, Ki TaeWu, JianyongGoltsov, AlexeyNamiesnik, JacekWu, Jin-YiGomes, Ana P.Nandha Premnath, PadmavathyWu, Li-ChenGómez, Ana MaríaNanjo, FumioWu, Ming-JiuanGómez, RodolfoNapoleão, Thiago HenriqueWu, Ming-JungGoncalves, Luis MoreiraNardi, MonicaWu, NickGonçalves, M. Sameiro T.Nasri, RimWu, Pao-ChuGonçalves, OlivierNassir, FatihaWu, Quan-XiangGonda, SándorNatalello, AntoninoWu, Sheng-ChiGong, JunboNaumova, Anna V.Wu, Shih-HsiungGong, XingchuNavarra, MicheleWu, ShihuaGonzales, Gerard BryanNavarro, Daniela M. A. F.Wu, Shin-TsonGonzález, Concepción C.Nawrocka, AgnieszkaWu, Tung-KungGonzález, Florenci V.Nazaré, MarcWu, Tzong-YuanGonzález Domínguez, RaúlNazarewicz, RafalWu, Wen-HueyGonzález-Andrade, MartínNazaruk, JolantaWu, XiaofengGonzález-Burgos, Elena MariaNazzaro, FilomenaWu, XiaoyuGonzález-Díaz, HumbertoNebigil, Canan G.Wu, Yang-ChangGonzález-Díaz, OscarNedachi, TakuWu, ZhenghongGonzález-Fernández, A.Neely, Brian J.Wu, ZhenqiangGonzález-Muñiz, RosarioNegri, FabriziaWu, ZhishengGonzález-Sarrías, AntonioNeilson, AndrewWu, Alan HbGorini Da Veiga, AnaNekkanti, VijaykumarWulff, Jeremy E.Gorman, Christopher B.Nekongo, Emmanuel E.Wyman, IanGorman, Gregory SNemoto, KiyomitsuXavier, Nuno ManuelGorska, MagdalenaNemykin, VictorXi, GuifaGórska, SabinaNenadis, NikolaosXi, WeixianGossage, R. A.Nerín, CristinaXia, WeimingGoto, YukiNesmelova, Irina V.Xie, ChongGotor-Fernández, VicenteNesterov, DmytroXie, GuoxiangGouin, SebastienNeumann, TerrenceXie, JinghangGoulas, VlasiosNeunert, GrazynaXie, LimingGoulet, CharlesNeureiter, DanielXie, QianGouws, ChrisnaNevárez-Moorillón, Guadalupe VirginiaXie, YingGovind, NiranjanNeves, M. Graça P.M.SXiming, WangGozzo, FrancoNewman, DavidXin, DongyueGrace, Dr. MaryNewmaster, Prof. Steven G.Xing, JunjiGraf, MichaelNg, Li FangXiong, YanGraikou, DiaNg, Tzi BunXu, BaoshanGramza-Michałowska, AnnaNgo, Huynh ThienXu, JieGranado, M. DoloresNguyen, Minh ThoXu, PeishengGrandfils, C.Nguyen, TuyenXu, QiongmingGrant, GeorgeNicklaus, Marc C.Xu, WangGrassi, AlfonsoNicoletti, RosarioXu, XingshunGravel, EdmondNielsen, HenrikXu, XudongGray, Christopher A.Nielsen, Line HagnerXu, XuemingGrée, RenéNielsen, Mogens BrøndstedXu, YanGreen, DonNielsen, Peter E.Xu, Yi JunGreenwood, DavidNiemantsverdriet, J. W.Xu, YunGreffrath, WolfgangNieuwenhuys, BernardXuan, Tran DangGressent, FrédéricNihei, Ken-ichiYaghmur, AnanGrider, ArthurNiikura, KenichiYahia, L’HocineGrieco, FrancescoNikas, Spyros P.Yajima, ShunsukeGrigalevicius, SauliusNikitenko, Sergey I.Yamada, KiyofumiGrilli, SeleneNikolakakis, IoannisYamada, SohsukeGrkovic, TanjaNippe, MichaelYamada, YusukeGrooten, JohanNishikata, TakashiYamagishi, TakehiroGross, AaronNishiwaki, HisashiYamaguchi, KanjiGross, HaraldNishizawa, SeiichiYamaguchi, MasahikoGrossini, ElenaNitulescu, George MihaiYamaguchi, YoshihiroGrosso, ClaraNiu, Xiao D.Yamaki, KohjiGrosvenor, AndrewNiwa, ToshioYamaki, KouyaGrøtli, MortenNiwano, YoshimiYamamoto, YasunoriGrovel, OlivierNo, Joo HwanYamanaka, T.Groysman, StanislavNobili, StefaniaYamashita, TeruhitoGruber, ChristianNoël, TimothyYamauchi, SatoshiGruber, JanNoge, KojiYamazaki, HiroyukiGrumet, RebeccaNoh, Jeong SookYan, DongqingGruselle, MichelNolte-’t Hoen, Esther N. M.Yan, HongGrynkiewicz, GrzegorzNomikos, TzortzisYan, XuehaiGrynszpan, FlavioNomura, YoshihiroYan Long, GuGrzegorczyk-Karolak, IzabelaNonami, AtsushiYanagita, Ryo C.Gu, JingkaiNonappa, NonappaYanase, EmikoGu, JunfengNormand, PhilippeYang, ChengGu, WenyiNorvez, SophieYang, Chia-NingGu, ZhenNotarangelo, FrancescaYang, Chi-ChiangGuan, YongguangNotni, JohannesYang, Ding-IGudiminchi, RamaNovella, SusanaYang, Ding-YahGuerrero, AntonioNovello, VittorinoYang, GuangdongGuerrero, JoséNovy, PavelYang, Hsiao-ChingGuetschow, MichaelNowak, RenataYang, Hung-WeiGugliandolo, ConcettaNowak, SaschaYang, JinsongGuiamet, PatriciaNowak-Sliwinska, PatrycjaYang, LiuGuibal, EricNowicka, Anna MariaYang, MinGuidotti, MatteoNowicki, JanuszYang, Pei-MingGuilhon, Giselle Maria S. P.Ntalli, Nikoletta G.Yang, QianruGuillou, HervéNtie-Kang, FideleYang, Seung OkGuiné, Raquel P. F.Nuhn, LutzYang, Si KyungGulati, PankajNycz, Jacek EYang, Wei-DongGulati, VandanaNyokong, TebelloYang, Wei-hsiungGunia-Krzyżak, AgnieszkaNyulász, LászlóYang, Wen-binGunness, PurnimaO’Connell, Mary A.Yang, XiuweiGuo, FangObenland, DavidYang, YaoGuo, FeiOberholster, AnitaYang, Ya-TangGuo, FenghaiObied, HassanYang, Yu-ChiaoGuo, HongchaoOda, AkifumiYang, Yu-liangGuo, HuiminOda, TatsuyaYang, ZhenGuo, Jia-HsinOdell, LukeYang, ZhijunGuo, PuO’Doherty, GeorgeYao, Ching FaGuo, Yuan QiangOestreich, MartinYao, ZhiliGuo, ZhengOga, Enoche F.Yao, Zhong-KeGupta, AnkitOgawa, AkiyaYaoita, YasunoriGurevich, Eugenia V.Ogawa, NorikoYarmolinsky, LudmilaGurramkonda, ChandrasekharOgawa, ShojiroYasukawa, KiyoshiGustavsson, ThomasOgihara, TakuoYau, SonataGutiérrez Martín, SantiagoOgino, ShujiYe, DeyongGutiérrez-Juárez, RogerOh, Jae-MinYe, ZhaoyangGutiérrez-Uribe, JanetOh, Jung HwanYe, ZhibinGutmann, TorstenOh, Kyung WhaYedery, Roshan D.Guzik, UrszulaOh, SeikwanYeh, Chih-koGuzmán, FannyOh, Sei-RyangYeh, Jan-yingGuzzo, FlaviaOh, Won KeunYeh, Ming-Chang P.Gynther, MikkoOhisa, SatoruYeh, PamelaHabata, YoichiOhmura, KoichiroYeh, Shu-LanHabaue, ShigekiOhnishi, MasatoshiYen, Chia-HungHabiba, KhaledOhshima, TatsuyaYen, Feng-LinHachiya, AkiraOhshima, TomokoYeong, Wai YeeHacker, Michael C.Ohta, MasayaYerabolu, Jayasudhan ReddyHadjikakou, Sotiris K.Ohta, ShinjiYiannakopoulou, EugeniaHadjipavlou-Litina, DimitraOhta, YoshijiYim, ChangyongHagenlocher, YvonneOikawa, AkiraYin, GuoweiHahn, EkkehardtOikawa, HideakiYin, Mei-chinHaider, NorbertOjika, MakotoYin, ShengHalabalaki, MariaOkada, TaketoYin, ZhiweiHaleagrahara, NagarajaOkafor, FlorenceYlijoki, Kai E. O.Hallett-Tapley, GenieceOkamura, NobuyukiYokomatsu, TsutomuHallsworth, J. E.Okazawa, AtsushiYokosuka, AkihitoHamad-Schifferli, KimberlyOkino, TatsufumiYoo, Eun JeongHamanishi, JunzoOkumura, TadayoshiYoo, Jung SunHamberger, BjoernOledzka, EwaYoon, Do-YoungHamon, Jean-RenéOlenyuk, Bogdan Z.Yoon, In-SooHan, JaehongOliva, RominaYoon, Kee DongHan, JianlinOliveira, M. Beatriz P. P.Yoon, Moon-YoungHan, Quan-BinOliveira, P.A.Yoon, Tae-HoonHan, Sang-baeOliveira Rocha, Hugo AlexandreYoshida, JunHan, Sang-MiOliverio, ManuelaYoshida, SuguruHan, Se JongOlivieri, GiuseppeYoshida, TakashiHan, XiaoyuanOlson, KennethYoshihito, YokoyamaHan, Zhengxu S.Olson, Mark A.Yoshikawa, MasaakiHanaor, DorianOmae, IwaoYoshimoto, MakotoHancock, Robert D.Omar, Syed HarisYoshimura, YuichiHandy, ScottOmonov, TolibjonYoshioka, NaokiHanhineva, KatiOmoruyi, FelixYou, LiangHano, ChristopheOmosa, Leonidah K.You, ZhongluHanschen, Franziska S.Onduka, ToshimitsuYoung, Jette F.Hansen, EspenOnnis, ValentinaYoung, SarahHansen, NielsOoyama, YousukeYu, ChengzhongHanson, James R.Opatz, TillYu, DonghongHanson, Robert N.Operamolla, AlessandraYu, GuangliHanusa, TimothyOrecchio, SantinoYu, HuaHao, HongdongOrtega, JoséYu, JinhongHao, XiaojuanOrtega, Maria TeresaYu, MeihuaHarada, NobuyukiOrtinski, Pavel I.Yu, Shi-ShanHarbourne, NiamhOrtiz, Fernando LópezYu, ShouyunHardeland, RudigerOrtiz Sainz de Aja, AlfredoYu, WenquanHare, Joshua M.Ortiz-Mellet, CarmenYu, YinghuaHarenberg, J.Ortiz-Urquiza, AlmudenaYuan, CSHarpke, DörteOsborn, HelenYuan, YaxiaHarris, PhilipOsbourn, AnneYuan, YouyongHarrison, Anna L.Ospina Millan, ClaudiaYuan, ZhaoHarrowfield, JackOstareck, Dirk H.Yuba, EijiHarwood, TimOthman, Zeid A. AlYue, PatrickYingKitHasegawa, MorifumiOttonelli, MassimoYumoto, HiromichiHasegawa, UraraOyewumi, MosesYun, Soon-IlHashimoto, MakotoOzawa, FumiyukiYun, Young SookHashimoto, NaotoOzeki, YasuhiroZabetakis, LoannisHashmi, A. Stephen K.Pacifico, SeverinaZabotina, OlgaHassan, Hassan M. A.Padmalayam, IndiraZaccheria, FedericaHassan, MohamedPadovan, AmandaZaferanloo, BitaHassan, Yousef I.Paduch, RomanZagotto, GiuseppeHatae, NoriyukiPaeng, Ki-JungZakrzewicz, DariuszHatano, TsutomuPagliaro, PasqualeZakrzewski, JerzyHattori, Haroldo T.Paik, Hyun-dongZamaratskaia, GaliaHaug, Bengt ErikPak, Sok CheonZambrowicz, AleksandraHautbergue, GuillaumePakulski, ZbigniewZamora, FernandoHay, Michael P.Pal, RiturajZanardi, ChiaraHayashi, TakashiPalacios, SaraZanetti, MichelaHayes, Douglas G.Pałasz, AleksandraZannatal, FerdousHaystead, TimothyPalazon, JavierZaprutko, LucjuszHe, BinPalazzo, EnzaZard, Samir Z.He, ChengweiPalenzuela López, José AntonioZarrelli, ArmandoHe, GuPaller, ChanningZarubaev, VladimirHe, JunPalm, Gottfried J.Zawilska, Jolanta B.Hearn, Michael J.Palomar, ManuelZehnacker-Rentien, AnneHedtrich, SarahPalumbo, AnnaZeiner, MichaelaHeery, David M.Pan, Tai-longZeiner, TimHehlgans, StephaniePan, Tzu-MingZeller, Wayne E.Heidary, Damoon Sohrabi BabaPan, Wei-PingZeng, DexingHeinrich, TimoPanacek, AlešZeng, HuaqiangHeiskanen, JuhaPanaro, Maria AntoniettaZeng, JieHeitz, ValériePanek, JarosławZeng, WenbinHelena, SovovaPaneth, AgataZeng, XiaomingHelesbeux, Jean-JacquesPanfoli, IsabellaZerbetto, FrancescoHelm, RichardPantazis, Dimitrios A.Zgoła-Grześkowiak, AgnieszkaHemming, KarlPanuwet, ParinyaZhang, ChiHenderson, BillPanzella, LuciaZhang, DaweiHenderson, Luke C.Paola, Luisa DiZhang, DengsongHendrich, SuzannePaola, StiusoZhang, FanHennig, AndreasPaoli, PaolaZhang, GeHensela, AndreasPaoli, PaoloZhang, GuojianHerdewijn, PietPaolone, AnnalisaZhang, GuolinHernández, FranciscaPapadimitriou, VassilikiZhang, HongHernández, Pablo E.Papageorgiou, MariaZhang, JingJingHernández-Ledesma, BlancaPapaioannou, DionissiosZhang, JinsongHerraiz, T.Papetti, AdeleZhang, JunboHerrera, Raquel P.Papini, AlessioZhang, JunyingHerrero, LauraParé, Paul W.Zhang, JunzengHerrmann, FabianPark, Chung GyooZhang, KaiHerrmann, MartinPark, Hee-juhnZhang, KunHervés-Beloso, PabloPark, Il-KwonZhang, LiangliangHettiarachchi, D. S.Park, Jeong HoZhang, LongheHewitson, PeterPark, Ji-yeonZhang, QiangHickman, James J.Park, Jong KukZhang, QinghaiHidalgo, DoloresPark, Jong-SeokZhang, QingwenHidalgo Herrador, José MiguelPark, Joo-inZhang, ShaozhongHidari, KazuyaPark, Nam-GyuZhang, ShuHideg, KálmánPark, Seung-ChunZhang, WeiHiejima, YusukePark, Soo-JinZhang, XiquanHiguchi, AkonPark, WansuZhang, YanminHingorani, DinaPark, Yong SeekZhang, YixiangHintermann, LukasParkinson, Don-RogerZhang, YonghongHirakawa, HidekiParmeggiani, FabioZhang, YuhangHirakawa, KazutakaParolini, CinziaZhang, YuqingHirano, TomoyaParpinello, Giuseppina PaolaZhang, Z.Hirao, IchiroParsons, J. G.Zhang, ZhanhuiHirasawa, NoriyasuParvez, MohammadZhang, ZhipingHirayama, FumitoshiPaschalidis, Konstantinos A.Zhang, ZhuoHirche, ChristophPascual, AlbertoZhao, HongboHirota, TakashiPasinetti, GiulioZhao, JincaiHiroyuki, TanakaPasini, DarioZhao, Jin-HaoHirsch, E.Pasini, LuigiZhao, LiangHo, Chi-TangPasqua, GabriellaZhao, YanHo, Su-ChenPasqua, TeresaZhao, YanchuanHodge, David R.Passalaqua, KarlaZheng, GaoweiHofer, TimPassamonti, SabinaZheng, Qi-HuangHoff, Bård HelgePassarella, DanieleZheng, ShilongHoffman, Angela M.Passey, Samantha L.Zheng, WeijieHofmann, BettinaPastor, JoaquinZheng, WeijunHofmann, JohannPastoriza, IsabelZheng, YingHofmann, UtePatel, HardikkumarZhong, GuohuaHohenegger, MartinPathuri, GopalZhong, HaizhenHojjat-Farsangi, MohammadPatruczynik, AnnaZhong, TuhuaHolder, AlvinPauer, WernerZhou, Cong-YingHolley, Richard A.Paulsen, Berit SmestadZhou, Hai-BingHolm, RenePaululat, ThomasZhou, JiajingHolst, OttoPautz, AndreaZhou, LigangHolt, CarlPavagadhi, ShrutiZhou, YifaHolzmann, NicolePavic, ValentinaZhu, CanHong, GuosongPavlidis, IoannisZhu, Junwei J.Hong, YonggeunPavon-Djavid, GracielaZhu, Prof. Dr. ChangjinHong, YuningPavone, Luigi MicheleZhu, XingyuanHopkins, SamanthaPeana, MassimilianoZidorn, CHöppener, StephaniePeczuh, MarkZiegler, ThomasHorbowicz, MarcinPedatella, SilvanaZielińska, SylwiaHori, YuichiroPedretti, AlessandroZielińska-Pisklak, Monika A.Horowitz, John D.Pedro, Maireles-TorresZielonka, JacekHorrigan, Louise A.Pélinski, LydieZiemba, BarbaraHorvath, David P.Pellecchia, MaurizioZimmer, ReinholdHosoya, TakamitsuPellis, AlessandroZiogas, DimosthenisHossain, Mohammad BillalPeltonen, LeenaZiółek, MarcinHou, AiqinPelus, LouisZi-Sheng, LuoHou, AnfuPeluso, GianfrancoZitko, JanHou, Duen-renPenczek, StanislawZłotek, UrszulaHou, HarveyPeneva, KalinaŻołnowska, BeataHou, ZhengqingPenfold, ChristopherZong, Xu-xiaoHoubracken, IsabellePeng, JinyongZoranovic, TamaraHouen, GunnarPeng, RobertZordoky, BeshayHouston, DouglasPeng, RuoguZotchev, SergeyHsia, Shih-MinPeng, Wen-HuangZou, TaotaoHsiao, GeorgePepper, Michael S.Zoumpoulakis, PanagiotisHsieh, ChangweiPeragón, JuanZrenner, Rita MariaHsieh, Jen-chienPeral, JoséZrnčić, MirtaHsieh, Jung-FengPereira, FlorbelaZuo, LiHsieh, Pei-WenPereira, José AugustoZupko, IstvanHsin, Kun-YiPereira, LeonelZygmunt, MałgorzataHsu, Shan-huiPereira, Maria G.Zylinska, LudmilaHu, AnmingPereira, Olívia R.


